# *Snora54* negatively regulates self-renewal of intestinal stem cells and gut regeneration via suppression of Notch2 signaling

**DOI:** 10.1126/sciadv.adv4725

**Published:** 2025-05-23

**Authors:** Jiahang Zhang, Hui Guo, Yuwei Xu, Zhen Xiong, Ying Du, Pingping Zhu, Zusen Fan

**Affiliations:** ^1^ State Key Laboratory of RNA Science and Engineering, State Key Laboratory of Epigenetic Regulation and Intervention, Institute of Biophysics, Chinese Academy of Sciences, Beijing, China.; ^2^ University of Chinese Academy of Sciences, Beijing, China.; ^3^ School of Life Sciences, Zhengzhou University, Zhengzhou, China.

## Abstract

The self-renewal of intestinal stem cells (ISCs) is essential for maintaining intestinal homeostasis and ensuring regeneration of the intestinal epithelium. However, whether small nucleolar RNAs participate in the regulation of ISC self-renewal remains unclear. Here, we identified a small nucleolar RNA (*Snora54*) that was highly expressed in the nucleolus of ISCs. *Snora54* knockout enhanced the self-renewal capacity of ISCs and intestinal regeneration. Mechanistically, in a steady state, highly expressed *Snora54* anchored the nucleolar protein Lyar in the nucleolus of ISCs, preventing Lyar from translocation into the nucleoplasm. Thereby, Lyar failed to recruit on the *Notch2* promoter region in the nucleoplasm to promote *Notch2* transcription, leading to suppression of ISC self-renewal. By contrast, with deletion of *Snora54*, Lyar translocated to the nucleoplasm of ISCs where it enriched on the *Notch2* promoter to initiate its transcription resulting in the activation of Notch2 signaling pathway. Therefore, *Snora54* negatively regulates self-renewal of ISCs and gut regeneration via suppression of Notch2 signaling.

## Teaser

Snora54 inhibits Notch2 signaling by regulating the nuclear localization of Lyar, thereby negatively regulating ISC self-renewal.

## INTRODUCTION

Most intestinal epithelial cells survive only a few days before being shed into the intestinal lumen from the tip of the villi. This high turnover rate is achieved through the rapid proliferation of intestinal stem cells (ISCs) located at the base of crypts, which generate transient amplifying (TA) cells (*1*) and consequently differentiate into various intestinal cell types, including enteroendocrine cells, goblet cells, tuft cells, Paneth cells, and others (*2*–*4*). Currently, ISCs are considered to comprise two main populations: crypt base columnar cells marked by Lgr5 and a loosely defined population of quiescent cells located at the +4 cell position (*5*). The self-renewal capacity of ISCs plays a crucial role in maintaining intestinal homeostasis and in the repair of intestinal damage. In vitro, the development of single ISCs into intestinal organoids can replicate the regenerative capacity of the intestinal epithelium (*6*–*8*). The function of ISCs is sustained by multiple stemness-related signaling pathways, including Wnt (*9*), Notch (*10*, *11*), Hippo (*12*), bone morphogenetic protein (*13*), and Hedgehog (*14*) pathways. Although there have been numerous studies on these signaling pathways, the mechanisms that regulate these pathways in the self-renewal and differentiation of ISCs still remain elusive.

Small nucleolar RNAs (snoRNAs) are a class of small, single-stranded noncoding RNAs, typically ranging from 60 to 300 nucleotides in length (*15*). Most snoRNAs are localized in the nucleolus, where their classical functions involve forming small nucleolar ribonucleoproteins by binding to proteins, thereby facilitating the posttranscriptional modifications and maturation of ribosomal RNA (rRNA) (*16*). However, an increasing body of researches indicates that snoRNAs can exert diverse functions through various nonclassical pathways. For example, *SNORD50A* and *SNORD50B* are localized in the cytoplasm and directly interact to inhibit K-Ras, thereby suppressing tumor development (*17*). *SNORD88B* binds to Werner Syndrome Protein (WRN) in the nucleolus to inhibit its entry into the nucleoplasm, leading to suppression of the Hippo signaling pathway (*18*). *SNORA13* directly interacts with RPL23 to disrupt its binding to the 60*S* ribosomal subunit, promoting p53-mediated cellular senescence (*19*). However, how snoRNAs modulate ISC functions remains unclear.

Lyar is a zinc finger protein that is localized in the nucleolus. It has been reported that Lyar is a protein capable of shuttling between the nucleolus and the nucleoplasm. In the nucleolus, Lyar promotes biosynthesis of rRNAs (*20*, *21*), while in the nucleoplasm, it has transcription factor activity (*22*). For example, in erythroid progenitor cells, Lyar can bind to Prmt5 on the promoter regions of Hbg1 and Hbg2 to inhibit their expression (*23*). Lyar also interacts with the promoter region of Fscn1 to promote its expression, modulating the downstream fatty acid metabolism in colorectal cancer cells (*24*). In addition, Lyar can recruit Brd2 protein to chromatin and subsequently suppress Nanog transcription to regulate cell differentiation (*25*). However, up to date, there are no studies on the role of Lyar in ISC biology.

The Notch signaling pathway is one of the most classic stem cell–related signaling pathways. In ISCs, Notch receptors (primarily Notch1 and Notch2) engage with Notch ligands (mainly Dll1 and Dll4) secreted by Paneth cells to activate downstream signaling, maintaining self-renewal for the stem cell pool and guiding differentiation of progenitor cells (*26*). When Notch signaling is up-regulated, ISCs tend to favor rapid self-renewal capacity and differentiation into TA cells, leading to differentiation into intestinal absorptive cells and promotion of epithelial regeneration. In contrast, when Notch signaling is down-regulated, the numbers of ISCs and TA cells decrease, resulting in production of secretory lineage cells (*27*–*29*). These findings indicate that the Notch signaling pathway plays a critical role in the maintenance of ISC stemness and intestinal homeostasis. Here, we identified a conserved snoRNA *Snora54* that was highly expressed in the nucleolus of ISCs. *Snora54* negatively regulates self-renewal of ISCs and gut regeneration via suppression of Notch2 signaling pathway.

## RESULTS

### *Snora54* is highly expressed in ISCs

**Fig. 1.**
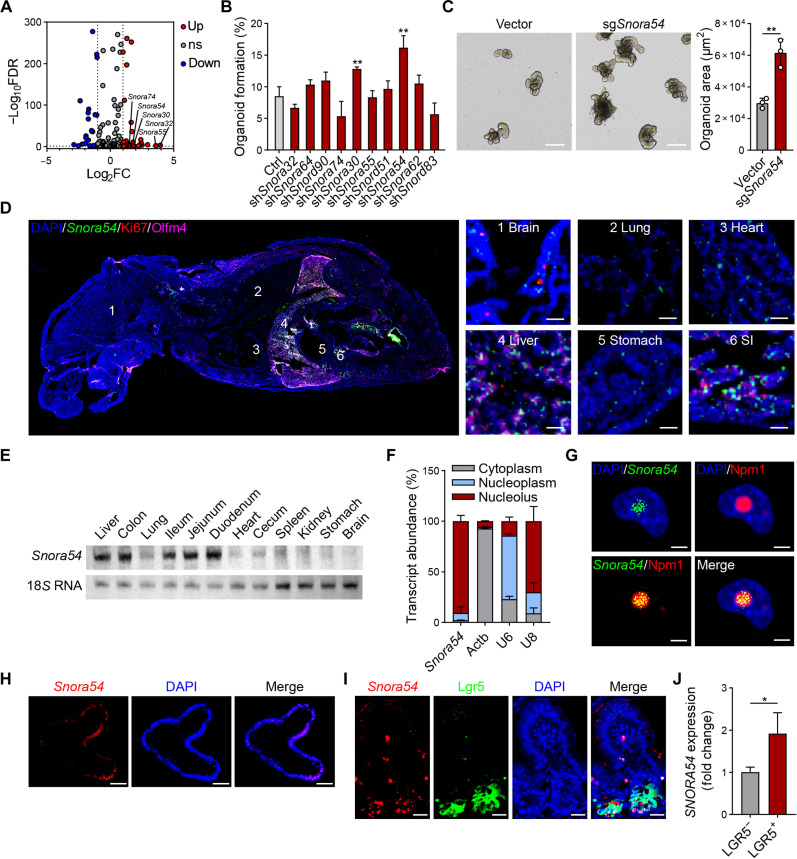
*Snora54* is highly expressed in the nucleolus of ISCs. (**A**) Volcano plot of differentially expressed snoRNAs in crypts and epithelial cells from C57BL/6 mice. ns, not significant. (**B**) Small intestine crypts were isolated from C57BL/6 mice for organoid formation. Ten up-regulated snoRNAs were depleted in organoids with shRNA strategy, followed by organoid formation. *n* = 3 independent experiments. Organoid formation ratios are shown as means ± SD. **P < 0.01 by two-tailed Student’s *t* test. (**C**) Images of intestinal organoids cultured under *Snora54* deletion and control. *n* = 3 separate organoid assays were performed. Scale bars, 200 μm. (**D**) One-week-old mice were euthanized for longitudinal sections. A global view of section is shown in left panel, and indicated tissues are shown to the right. 1, brain; 2, lung; 3, heart; 4, liver; 5, stomach; 6, SI (small intestine). Scale bars, 50 μm. (**E**) Northern blot analysis of *Snora54* in different tissues of 2-month-old mice, 18*S* RNA served as a loading control. Three independent experiments were performed with similar results, and representative experiments are shown. (**F**) Relative distribution of *Snora54* in mouse ISCs determined by qRT-PCR in different cell fractions. *n* = 3 independent experiments. Data are shown as means ± SD. (**G** to **I**) RNA FISH of *Snora54* in CT26 cells (G), small intestine organoid (H), and small intestine tissue (I). Scale bars, 5 μm (G) and 50 μm [(H) and (I)]. (**J**) Expression of *SNORA54* in human LGR5^−^ and LGR5^+^ cells was detected by qRT-PCR. *n* = 3 independent experiments. Results are shown as means ± SD. *P < 0.05 by two-tailed Student’s *t* test.

To investigate the effect of snoRNAs on the regulation of ISCs, we extracted crypts and epithelial cells from C57BL/6 mice and performed snoRNA sequencing. Top 10 snoRNAs highly expressed in the crypts were selected for subsequent analysis (Fig. 1A). Intestinal organoids are derived from ISCs and have microstructures similar to those of intestinal tissues, which manifests the capacity for intestinal tissue regeneration and homeostasis maintenance (*7*). We used short hairpin RNA (shRNA) to deplete specific snoRNAs in ISCs and conducted organoid formation experiments. We found that depletion of *Snora54* most markedly augmented formation of organoids (Fig. 1B and fig. S1A). Parallelly, *Snora54* knockout promoted organoid formation and growth (Fig. 1C). *Snora54* is located between exons 8 and 9 of *Nap1l4* gene on mouse chromosome 7 (for humans, chromosome 11) (fig. S1, B and C), having a classic H/ACA box structure (fig. S1D). In addition, *Snora54* showed a high degree of conservation among mammals (fig. S1E). We next determined the localization of *Snora54* in various tissues using RNA fluorescence in situ hybridization (FISH) and quantitative reverse transcription polymerase chain reaction (qRT-PCR) assays. We found that *Snora54* was highly expressed in small intestines, colons, and livers (Fig. 1D and fig. S1F). These observations were further validated by Northern blotting (Fig. 1E). Through subcellular fractionation, we noticed that *Snora54* was primarily located in the nucleolus (Fig. 1F) and further confirmed by FISH assay (Fig. 1G). In addition, *Snora54* was mainly localized in Lgr5^+^ cells (fig. S1G). FISH analysis revealed that *Snora54* was predominantly located at the budding regions of organoids (Fig. 1H) and the bottoms of crypts (Fig. 1I), which indicated that *Snora54* was distributed in the Lgr5^+^ ISCs. Consistently, *Snora54* was not located in the Ki67^+^ TA cells (fig. S1H). Last, we found that *SNORA54* was also highly expressed in human Lgr5^+^ ISCs (Fig. 1J). Collectively, these results indicate that *Snora54* is highly expressed in the intestinal ISCs and distributed in the nucleolus.

### *Snora54* knockout promotes the self-renewal of ISCs and intestinal regeneration

To explore the physiological role of *Snora54* in ISCs, we generated *Snora54* knockout (*Snora54*^−/−^) mice using CRISPR-Cas9 technology (fig. S2A). The deletion of a 159–base pair (bp) fragment containing *Snora54* was confirmed through genotyping and PCR (fig. S2B). *Snora54* was completely deleted in *Snora54*^−/−^ mice compared to littermate *Snora54*^+/+^ mice (fig. S2, C and D). In contrast, the expression of *Nap1l4*, the parental gene of *Snora54*, showed no apparent change between *Snora54*^+/+^ and *Snora54*^−/−^ mice by qRT-PCR (fig. S2E) and Western blotting (fig. S2F). In addition, depletion of Nap1l4 did not obviously affect organoid formation compared to empty vector control (fig. S2, G and H).

**Fig. 2.**
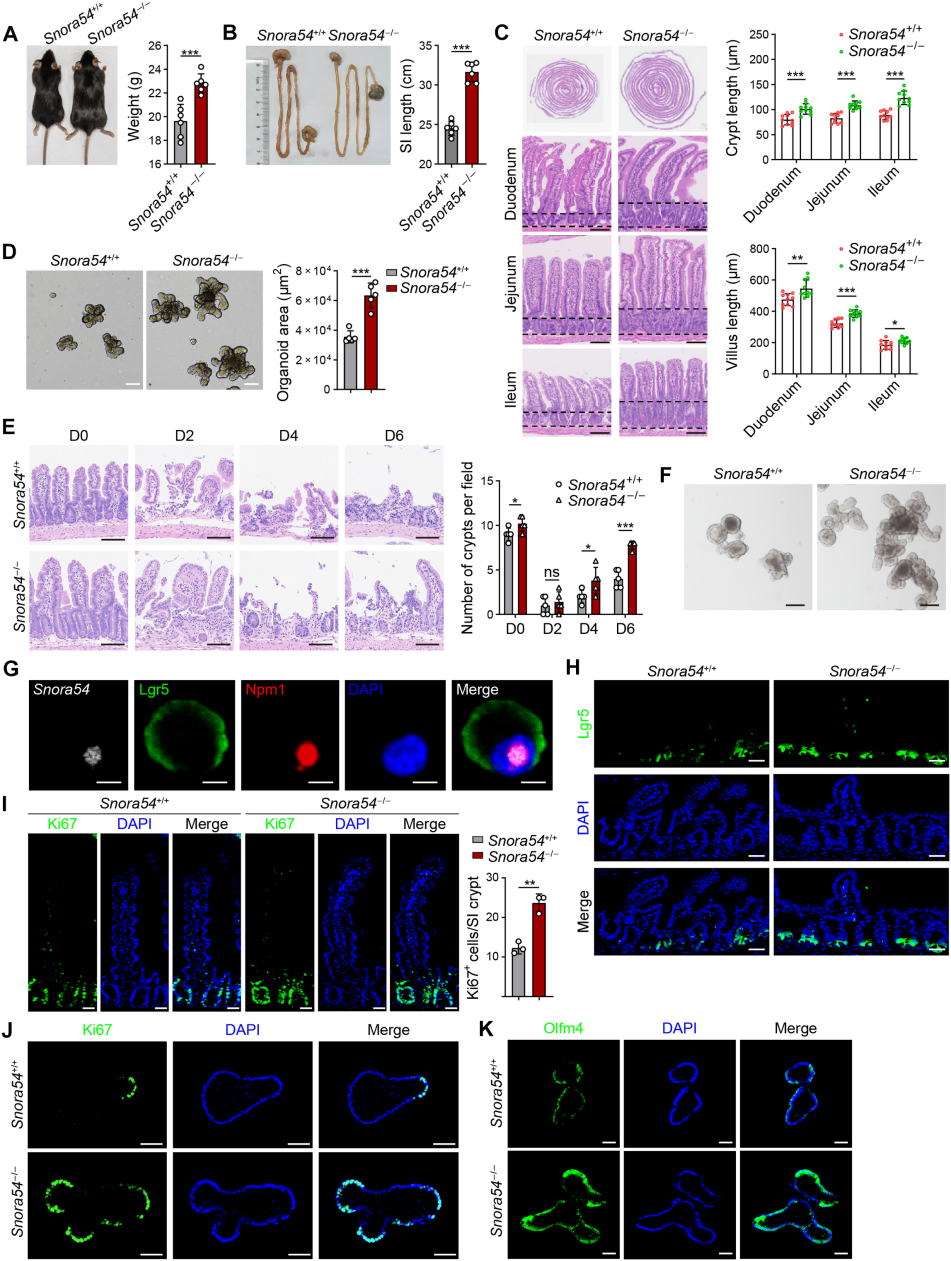
*Snora54* knockout promotes self-renewal of ISCs and gut regeneration. (**A**) Image of *Snora54*^+/+^ and *Snora54*^−/−^ mice. *n* = 6 mice were used. Body weight are shown as means ± SD. ***P < 0.001. (**B**) Small intestine image of *Snora54*^+/+^ and *Snora54*^−/−^ mice. *n* = 6 mice were used. Small intestine lengths are shown as means ± SD. ***P < 0.001. (**C**) Hematoxylin and eosin (H&E) staining of global view for small intestine from *Snora54*^+/+^ and *Snora54*^−/−^ mice. Length of crypts and villus were calculated as means ± SD. *P < 0.05, **P < 0.01, and ***P < 0.001. *n* = 10 fields of each group from six mice. Scale bars, 100 μm. (**D**) Organoids cultured from ISCs derived from *Snora54*^+/+^ and *Snora54*^−/−^ mice. Organoid areas per field (*n* = 5) are shown as means ± SD. ***P < 0.001. Scale bars, 100 μm. (**E**) H&E staining of small intestine tissues from indicated mice at different time points [day 0 (D0) to day 6] after 8-Gy radiation damage. Numbers of crypts were calculated as means ± SD. *P < 0.05 and ***P < 0.001. *n* = 5 fields were calculated for each group. Scale bars, 100 μm. (**F**) Quantification of organoid formation from *Snora54*^+/+^ and *Snora54*^−/−^ mice induced by radiation (8 Gy) on day 6. Scale bars, 100 μm. (**G**) RNA FISH of *Snora54* in ISCs. Scale bars, 5 μm. (**H**) Confocal microscopy of small intestine tissues from Lgr5-GFP; *Snora54*^+/+^ and Lgr5-GFP; and *Snora54*^−/−^ mice. Scale bars, 50 μm. (**I**) Ki67 staining in small intestinal tissues from *Snora54*^+/+^ and *Snora54*^−/−^ mice. Ki67^+^ cells per crypt (*n* = 3) are shown as means ± SD. **P < 0.01. Scale bars, 50 μm. (**J** and **K**) Ki67 (J) and Olfm4 (K) staining in *Snora54*^+/+^ and *Snora54*^−/−^ organoids. Scale bars, 50 μm.

We observed that *Snora54*^−/−^ mice showed an overt increase in body weight, as well as longer small intestines and colons compared to littermate *Snora54*^+/+^ control mice (Fig. 2, A and B, and fig. S3A). Furthermore, *Snora54*^−/−^ mice displayed increased crypt and villus lengths across all three regions of small intestines (duodenum, jejunum, and ileum) (Fig. 2C). Crypt lengths of colons also increased (fig. S3B). However, *Snora54*^−/−^ mice exhibited no apparent changes in other organs including lungs, livers, stomachs, hearts, spleens, and kidneys (fig. S3C). To determine whether the increased crypt and villus lengths enhanced intestinal absorptive capacity, we measured levels of glucose and nonesterified fatty acids in the feces of both *Snora54*^−/−^ and *Snora54*^+/+^ mice. We noticed that both glucose and nonesterified fatty acid levels were reduced in the feces of *Snora54*^−/−^ mice compared to those of *Snora54*^+/+^ mice, suggesting an improvement in intestinal absorptive capacity (fig. S3, D and E). We extracted crypt cells from both *Snora54*^−/−^ and *Snora54*^+/+^ mice for organoid formation assay. We observed that *Snora54*^−/−^ crypt cells developed organoids with faster rates compared with *Snora54*^+/+^ crypt cells (Fig. 2D). Given that ISCs have injury-induced plasticity and have the ability for self-regeneration (*12*), we then tested intestinal regeneration of *Snora54*^−/−^ mice. We subjected both *Snora54*^−/−^ and *Snora54*^+/+^ mice to 8 gray (Gy) of radiation to cause intestinal epithelial damage. Compared to *Snora54*^+/+^ mice, the crypt damage in *Snora54*^−/−^ mice repaired with faster rates over radiation injury (Fig. 2E). In addition, *Snora54*^−/−^ ISCs showed enhanced organoid formation capacity after irradiation injury compared to *Snora54*^+/+^ ISCs (Fig. 2F).

We next stained *Snora54* in Lgr5^+^ cells isolated from the intestinal crypts by FISH. We found that *Snora54* was localized in the nucleolus of Lgr5^+^ ISCs (Fig. 2G). We then crossed *Snora54*^−/−^ or *Snora54*^+/+^ mice with Lgr5–green fluorescent protein (GFP) mice, followed by intestine sectioning staining of offspring. We observed that numbers of Lgr5^+^ ISCs at the base of the crypts were remarkably increased in *Snora54*^−/−^ mice compared to *Snora54*^+/+^ mice (Fig. 2H). Consequently, Ki67 staining revealed that proliferating cells were also increased in the crypts of *Snora54*^−/−^ mice (Fig. 2I). In addition, *Snora54*^−/−^ organoids also displayed remarkable increase in proliferating cells compared to *Snora54*^+/+^ counterparts (Fig. 2J). Notably, the ISC maker Olfm4 was up-regulated in *Snora54*^−/−^ mouse organoids compared with *Snora54*^+/+^ counterparts (Fig. 2K). Together, *Snora54* deletion augments the self-renewal capacity of ISCs and intestinal regeneration.

### *Snora54* overexpression suppresses the self-renewal of ISCs and organoid formation

**Fig. 3.**
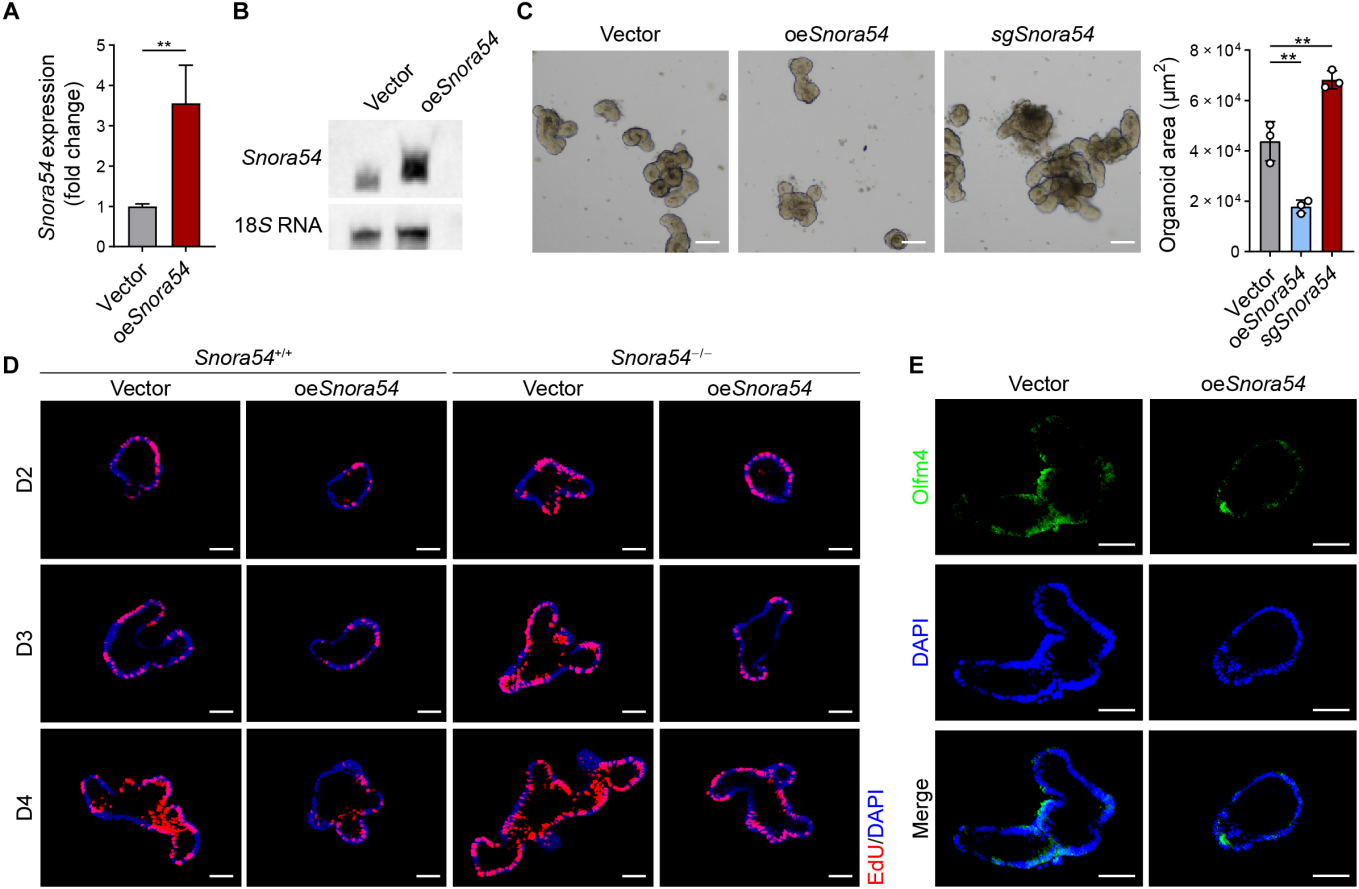
*Snora54* overexpression suppresses the self-renewal of ISCs. (**A**) Overexpression of *Snora54* in wild-type (WT) small intestine organoids was detected by qRT-PCR. Empty vector served as a control. *n* = 3 independent experiments. Results are shown as means ± SD. **P < 0.01 by two-tailed Student’s *t* test. (**B**) Northern blot analysis for *Snora54* expression in oe*Snora54* organoids. 18*S* RNA served as a loading control. (**C**) Organoid formation was conducted with *Snora54* knockout and *Snora54* overexpression in WT mouse organoids. Empty vector served as a control. Organoid areas per field (*n* = 3) are shown as means ± SD. **P < 0.01 by two-tailed Student’s *t* test in right panel. Scale bars, 50 μm. (**D**) EdU incorporation staining of organoids from *Snora54* overexpression crypts in *Snora54*^+/+^ or *Snora54*^−/−^ mice. *Snora54* overexpression crypts in *Snora54*^+/+^ or *Snora54*^−/−^ mice were collected for organoid formation, and 10 μM EdU solution was added to cultured organoids 2 hours before collection. Scale bars, 50 μm. (**E**) Olfm4 staining in *Snora54* overexpression organoids. Empty vector served as a control. Scale bars, 50 μm.

We next overexpressed *Snora54* in Lgr5^+^ ISCs and conducted organoid formation assay. Overexpression efficiency was determined using qRT-PCR (Fig. 3A) and Northern blotting (Fig. 3B). We found that *Snora54* overexpression markedly suppressed organoid formation, while organoids formed by *Snora54*^−/−^ ISCs were increased as a control (Fig. 3C). We then overexpressed *Snora54* in *Snora54*^−/−^ or *Snora54*^+/+^ organoids, followed by assessment of their proliferation capacity by ethynyldeoxyuridine (EdU) incorporation assay at days 2 to 4. We found that *Snora54* overexpression in *Snora54*^+/+^ organoids markedly inhibited organoid proliferation compared to empty vector–overexpressing counterparts (Fig. 3D). Consistently, *Snora54* overexpression in *Snora54*^−/−^ organoids also inhibited organoid proliferation, whereas *Snora54*^−/−^ organoids exhibited marked organoid formation (Fig. 3D). In addition, Olfm4 was down-regulated in *Snora54* overexpression mouse organoids compared with vector counterparts (Fig. 3E). Collectively, *Snora54* overexpression suppresses the self-renewal of ISCs and organoid formation.

### *Snora54* associates Lyar in ISC nucleolus

We wanted to explore how *Snora54* regulated ISC stemness. Given that the classical function of H/ACA box snoRNAs is to regulate pseudouridylation of rRNA (*16*), we determined whether *Snora54* regulated ISCs through its classical mechanism. *N*-cyclohexyl-*N*′-(2-morpholinoethyl)carbodiimide (CMC) can bind to the sites of RNA pseudouridylation and inhibit reverse transcription (*30*, *31*). We treated RNAs from both *Snora54* knockout and control cells with CMC, followed by qRT-PCR against their predicted pseudouridylation sites (fig. S4, A and B). We found that *Snora54* mediated pseudouridylation modifications at positions 3478 and 4221 of 28*S* rRNA (fig. S4, C and D). However, *Snora54* deletion did not affect expression of 28*S* rRNA (fig. S4E). To test whether *Snora54* regulated the stemness of ISCs with its classical function, we generated a mutant *Snora54* (mut*Snora54*) with two mutations at the pseudouridylation sites. We observed that mut*Snora54* was unable to catalyze pseudouridylation at respective sites of 28*S* rRNA (fig. S4, F to H). We then overexpressed both *Snora54* and mut*Snora54* in *Snora54*^−/−^ ISCs, followed by organoid formation assay. We found that overexpression of *Snora54* and mut*Snora54* in *Snora54*^−/−^ ISCs could inhibit organoid formation and growth with similar rates (fig. S4I). These results indicate that *Snora54*-mediated pseudouridylation is not involved in the regulation of ISC stemness.

**Fig. 4.**
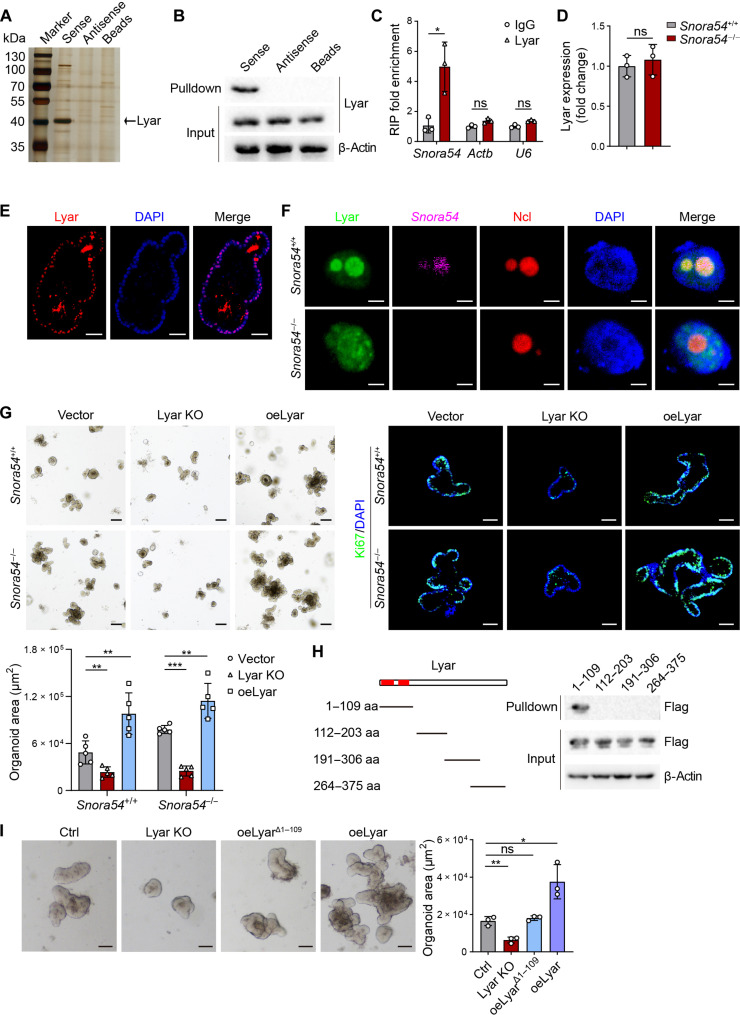
*Snora54* associates with Lyar in the nucleolus of ISCs. (**A**) Small intestine crypts from WT mice were lysed and incubated with biotin-labeled sense, antisense, or streptavidin magnetic beads. Eluted fractions were resolved by SDS–polyacrylamide gel electrophoresis (PAGE), followed by silver staining and mass spectrometry. Differential band to bind *Snora54* was identified as Lyar (black arrow). (**B**) Immunoblotting analysis of Lyar in RNA pull-down samples by *Snora54* sense, antisense, and control beads in mouse ISCs. (**C**) Small intestine crypts from WT mice were used for RIP assay and followed by qRT-PCR. *n* = 3 independent experiments. Results are shown as means ± SD. *P < 0.05. (**D**) Expression levels of Lyar in *Snora54*^+/+^ and *Snora54*^−/−^ ISCs were examined by qRT-PCR. *n* = 3 independent experiments. Results are shown as means ± SD. (**E**) Lyar staining in organoids cultured from WT mice. Scale bars, 50 μm. (**F**) Fluorescence imaging of *Snora54* and Lyar in *Snora54*^+/+^ and *Snora54*^−/−^ ISCs. Scale bars, 5 μm. (**G**) Organoid formation was conducted with Lyar knockout (Lyar KO) and Lyar overexpression (oeLyar) in *Snora54*^+/+^ and *Snora54*^−/−^ ISCs. Organoid areas per field (*n* = 5) are shown as means ± SD. **P < 0.01 and ***P < 0.001. Scale bars, 200 μm (top left) and 50 μm (top right). (**H**) Domain mapping analysis of *Snora54*-binding domains of Lyar protein. Different domains of Lyar protein were incubated with *Snora54*, followed by RNA pull-down assay and Western blotting. Red region, predicted binding domain; aa, amino acids. (**I**) Organoid formation was conducted with Lyar KO and oeLyar and Lyar-binding domain deletion overexpression (oeLyar^Δ1–109^). Organoid areas per field (*n* = 3) are shown as means ± SD. *P < 0.05 and **P < 0.01. Scale bars, 50 μm.

To investigate whether *Snora54* exerted its function through interacting protein candidates, we used biotin-labeled *Snora54* as a probe and performed RNA pull-down assays with mass spectrometry. Lyar, a zinc-finger protein, was identified as an associated candidate protein of *Snora54* (Fig. 4A and fig. S5, A and B). We then validated the interaction between *Snora54* and Lyar through immunoblotting (Fig. 4B) and RNA immunoprecipitation (RIP) assays (Fig. 4C). In addition, we found that the *Snora54* deletion did not affect the expression of Lyar (Fig. 4D). We observed that Lyar was localized in the nucleolus, which was colocalized with *Snora54* (Fig. 4E). Notably, in the presence of *Snora54*, Lyar was predominantly localized to the nucleolus. However, in the absence of *Snora54*, Lyar lose its nucleolar localization, distributing diffusely throughout the entire nucleus (Fig. 4F). We used *Snora31* probe as a positive control. *Snora31* probe fluorescence exhibited in both *Snora54*^+/+^ and *Snora54*^−/−^ ISCs (fig. S5C), indicating that *Snora54* probe is specific. In addition, we assessed accumulation of 47/45*S* pre-rRNA and levels of 28*S* rRNA in the absence of *Snora54* or Lyar. We observed that deletion of *Snora54* or Lyar did not change levels of 47/45*S* pre-rRNA and levels of 28*S* rRNA in ISCs, suggesting that Lyar is not involved in the pre-rRNA processing in ISCs (figs. S4E and S5, D to F). In addition, we noticed that *Snora54* deletion did not affect expression levels of Ncl or Npm1 (fig. S5, G and H). Moreover, fluorescent staining and electron microscopy results showed that *Snora54* deletion did not change nucleolar morphology (Fig. 4F and fig. S5I).

To further investigate the function of Lyar in the regulation of ISCs, we deleted or overexpressed Lyar and conducted organoid formation assay. We found that Lyar deletion remarkably inhibited organoid formation, while Lyar overexpression promoted organoid formation (Fig. 4G). In addition, in the absence of Lyar, *Snora54* or without *Snora54* did not affect organoid formation rates (Fig. 4G), suggesting that Lyar was involved in the *Snora54*-mediated stemness regulation of ISCs as a downstream partner. Through domain mapping assay, we identified that the N-terminal region of Lyar protein (1 to 109 amino acids) was essential for binding *Snora54*, which was consistent with our prediction (Fig. 4H and fig. S5, B and J). We next constructed a Lyar variant (Lyar^Δ1–109^) lacking amino acids 1 to 109 and overexpressed it in ISCs for organoid formation assay. We found that Lyar^Δ1–109^ variant did not affect expression of full-length Lyar and was unable to promote organoid formation (Fig. 4I and fig. S5K). Through fluorescence staining, we observed that Lyar^Δ1–109^ variant distributed in both nucleoplasm and in the nucleolus (fig. S5L). Together, these results indicate that Lyar is involved in the *Snora54*-mediated stemness regulation of ISCs.

### *Snora54* deletion causes nuclear translocation of Lyar to enrich on the Notch2 promoter for its transcription

**Fig. 5.**
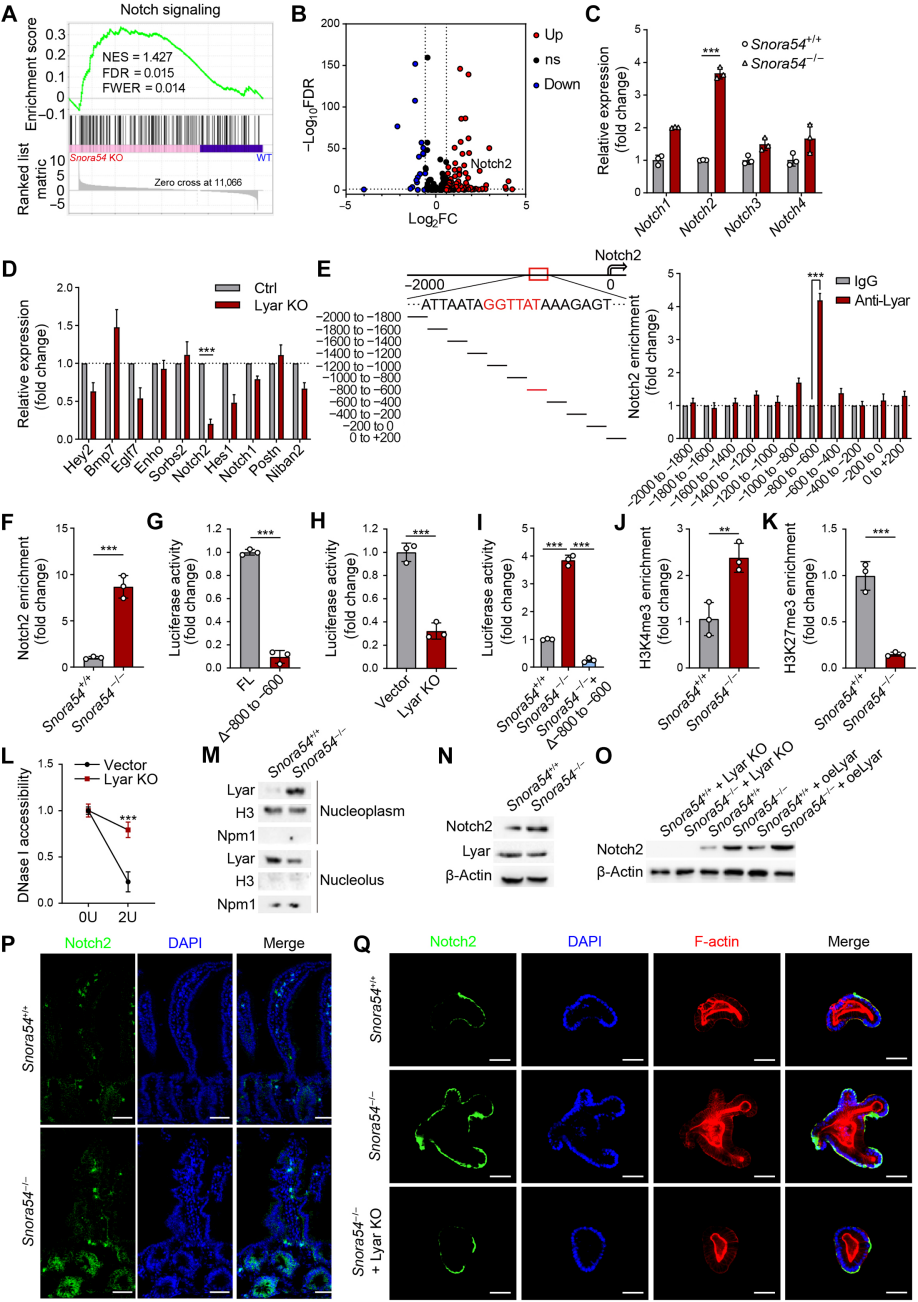
Lyar enriches onto the *Notch2* promoter to trigger its transcription. (**A**) GSEA analysis of Notch signaling pathway in *Snora54* KO and WT ISCs. NES, normalized enrichment score; FDR, false discovery rate; FWER, family-wise error rate. (**B**) Volcano plot of differentially expressed Notch-related gene in *Snora54* KO and WT ISCs. FC, fold change. (**C**) Expression levels of Notch receptors in *Snora54*^+/+^ and *Snora54*^−/−^ ISCs. *n* = 3. Results are shown as means ± SD. ***P < 0.001. (**D**) Expression levels of top 10 differentially expressed Notch-related genes from Lyar KO and WT organoids. *n* = 3. Results are shown as means ± SD. ***P < 0.001. (**E** and **F**) ChIP was performed in ISCs (E) and *Snora54*^−/−^ ISCs (F), followed by qRT-PCR. *n* = 3. Results are shown as means ± SD. ***P < 0.001. (**G** to **I**) Luciferase reporter assays were performed with Notch2 promoter-depletion (Δ−800 to −600) vectors (G), full-length Notch2 promoter in Lyar KO cells and vector cells (H), Notch2 promoter-depletion (Δ−800 to −600) vectors in *Snora54*^+/+^ and *Snora54*^−/−^ cells (I). *n* = 3. Results are shown as means ± SD. ***P < 0.001. (**J** and **K**) ChIP assay was performed using anti-H3K4me3 (J) or anti-H3K27me3 (K) with *Snora54*^+/+^ and *Snora54*^−/−^ ISCs. *n* = 3. Results are shown as means ± SD. ***P < 0.001. (**L**) DNase I accessibility assay was conducted using Lyar KO and vector organoids. *n* = 3. Results are shown as means ± SD. ***P < 0.001. 0U, 0 units; 2U, 2 units. (**M**) Immunoblotting analysis of Lyar in nucleolus and nucleoplasm components isolated from *Snora54*^+/+^ and *Snora54*^−/−^ ISCs. (**N**) Immunoblotting analysis of Notch2 and Lyar in *Snora54*^+/+^ and *Snora54*^−/−^ ISCs. (**O**) Immunoblotting analysis of Notch2 in oeLyar and Lyar KO organoids formed by *Snora54*^+/+^ and *Snora54*^−/−^ ISCs. (**P** and **Q**) Confocal microscopy of Notch2 in small intestine tissues (P) and organoids (Q) from *Snora54*^+/+^ and *Snora54*^−/−^ mice. Scale bars, 50 μm.

To further explore the molecular mechanism of *Snora54* in the regulation of ISCs, we conducted transcriptome analysis of ISCs isolated from *Snora54*^−/−^ and *Snora54*^+/+^ mice. Through gene set enrichment analysis (GSEA), the Notch signaling pathway was the most remarkably affected among stem cell signaling pathways (Fig. 5A and fig. S6, A to C). Moreover, Notch2 was most elevated in *Snora54*^−/−^ ISCs (Fig. 5, B and C). We then examined expression levels of Notch-related genes in Lyar knockout organoids by qRT-PCR. Consistently, Notch2 was most significantly reduced in Lyar deficient organoids (Fig. 5D). These data suggest that Lyar is involved in the regulation of Notch2 signaling pathway.

Previous studies demonstrated that Lyar preferentially binds to a DNA motif containing 5′-GGTTAT-3′ (*23*). We analyzed a 2-kb region upstream of the *Notch2* gene transcription start site and found that Lyar was enriched at the −800- to −600-bp region of the *Notch2* promoter, which contained the 5′-GGTTAT-3′ motif (Fig. 5E). We also found that deletion of *Snora54* promoted Lyar enrichment on the Notch2 promoter (Fig. 5F). In addition, deletion of the −800 to −600 region of the *Notch2* promoter markedly reduced luciferase activity (Fig. 5G). In parallel, Lyar deletion also decreased luciferase activity, suggesting that Lyar was involved in the regulation of *Notch2* transcription (Fig. 5H). In addition, deletion of *Snora54* markedly enhanced transcriptional activation of downstream genes. However, with deletion of the −800 to −600 region of the *Notch2* promoter, *Snora54* lost this role (Fig. 5I). Through chromatin immunoprecipitation (ChIP) assay, we observed that *Snora54* deletion enriched histone H3 lysine 4 trimethylation (H3K4me3) on the *Notch2* promoter in ISCs (Fig. 5J). In contrast, *Snora54* deletion decreased enrichment of H3K27me3 on the *Notch2* promoter in ISCs (Fig. 5K). Moreover, Lyar depletion remarkably augmented deoxyribonuclease I (DNase I) sensitivity of Notch2 promoter region (Fig. 5L).

We hypothesized that the regulation of Notch2 by *Snora54* might be related to the nuclear translocation of Lyar protein. We isolated nucleoplasm and nucleoli of ISCs and found that *Snora54* deletion caused translocation of Lyar protein into the nucleoplasm of ISCs (Fig. 5M). This observation indicates that deletion of *Snora54* led to translocation of Lyar from the nucleolus to the nucleoplasm. Notably, *Snora54* knockout caused elevated expression of Notch2 but had no effect on the expression of Lyar (Fig. 5N). By contrast, Lyar knockout markedly suppressed expression of Notch2, while overexpression of Lyar enhanced its expression (Fig. 5O). Notably, we observed that *Snora54*^−/−^ mice markedly increased Notch2 expression in intestinal crypts (Fig. 5P). Similar results were achieved in organoids derived by *Snora54*^−/−^ ISCs (Fig. 5Q). However, Lyar deletion in *Snora54*^−/−^ ISCs did not enhance Notch2 expression (Fig. 5Q), indicating that Lyar was a downstream regulatory factor in the *Snora54*-mediated stemness regulation of ISCs. Collectively, we conclude that *Snora54* deletion released Lyar into the nucleoplasm to recruit on the Notch2 promoter, leading to its transcription and expression in the ISCs.

### *Snora54* negatively regulates the stemness of ISCs via inhibition of Notch2 signaling pathway

**Fig. 6.**
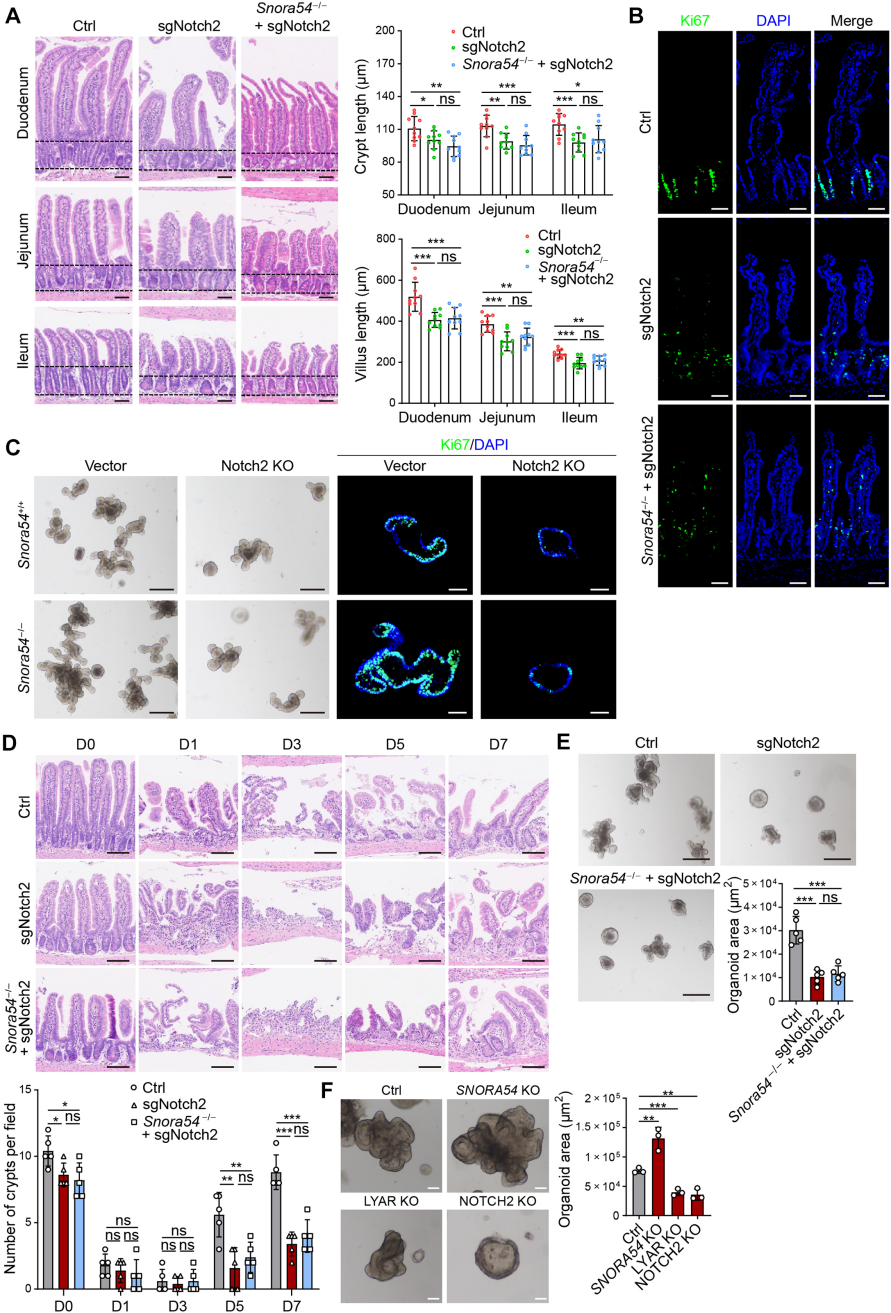
*Snora54* negatively regulates the stemness of ISCs via inhibition of Notch2 signaling. (**A**) H&E staining of small intestine from sgNotch2 and *Snora54*^−/−^;sgNotch2 mice. Average lengths of crypts and villus in a visual field from sgNotch2 and *Snora54*^−/−^;sgNotch2 mice are shown. Lengths of crypts and villus were calculated as means ± SD. *P < 0.05, **P < 0.01, and ***P < 0.001. *n* = 10 fields of each group from six mice were observed for length calculation and statistical analysis. Scale bars, 50 μm. (**B**) Representative images of Ki67 staining of small intestine from sgNotch2 and *Snora54*^-/−^;sgNotch2 mice. Scale bars, 50 μm. (**C**) Organoid formation was conducted with Notch2 knockout (Notch2 KO) in *Snora54*^+/+^ and *Snora54*^-/−^ ISCs. Scale bars, 200 μm (left) and 50 μm (right). (**D**) H&E staining of small intestine tissues from indicated mice at different time points (days 0 to 7) after 8-Gy radiation damage. Numbers of crypts were calculated as means ± SD. *P < 0.05, **P < 0.01, and ***P < 0.001. *n* = 5 fields were calculated for each group. Scale bars, 100 μm. (**E**) Quantification of organoid formation from intestinal crypts isolated from sgNotch2 and *Snora54*^-/−^;sgNotch2 mice induced by radiation (8 Gy) on day 5. *n* = 5 separate organoid assays were performed. Organoid areas were calculated as means ± SD. ***P < 0.001. Scale bars, 100 μm. (**F**) Human colon organoid formation was conducted with *Snora54* knockout (*SNORA54* KO), LYAR KO, and NOTCH2 KO. Organoid areas were calculated as means ± SD. **P < 0.01 and ***P < 0.001. *n* = 3 fields were calculated for each group. Scale bars, 50 μm.

We next generated Notch2-deficient mice (sgNotch2) using CRISPR-Cas9–mediated genome editing (*32*). We demonstrated that Notch2 were deleted in sgNotch2 mice by immunoblotting (fig. S7A). We observed that sgNotch2 mice displayed shortened crypts and villi (Fig. 6A). We also deleted *Notch2* in *Snora54*^−/−^ mice (*Snora54*^−/−^;sgNotch2) and found that *Snora54*^−/−^;sgNotch2 mice exhibited comparable phenotype to sgNotch2 mice (Fig. 6A). As expected, both sgNotch2 and *Snora54*^−/−^;sgNotch2 mice showed comparably reduced TA cells (Fig. 6B and fig. S7B). Wheat germ agglutinin (WGA) binds to vesicles in the intestinal tract to indicate secretory cells, including Paneth cells and goblet cells (*33*, *34*). We then stained WGA in small intestines and observed that WGA was markedly reduced in *Snora54*^−/−^ mice (fig. S7C), suggesting that *Snora54*^−/−^ mice caused a decreased number of secretory cells, which is consistent with the characteristics of down-regulated Notch signaling (*27*). These observations suggest that Notch2 is a downstream molecule of *Snora54* in the *Snora54*-mediated regulation of ISC stemness.

We also performed organoid formation assay. We found that both sgNotch2 and *Snora54*^−/−^;sgNotch2 ISCs showed reduced organoids and Ki67^+^ cells with comparable decreased rates (Fig. 6C). We then conduced irradiation and assessed gut regeneration ability in sgNotch2 and *Snora54*^−/−^;sgNotch2 mice. We noticed that both sgNotch2 and *Snora54*^−/−^;sgNotch2 mice displayed comparably impaired gut regeneration (Fig. 6D). Consistently, sgNotch2 and *Snora54*^−/−^;sgNotch2 ISCs derived from mice with day 5 postirradiation with 8 Gy displayed similar declined organoids (Fig. 6E). Given that *SNORA54* was highly expressed in human LGR5^+^ cells, we then conducted human colon organoid formation to validate whether *SNORA54* was involved in the regulation of self-renewal of ISCs. We found that deletion of *SNORA54* promoted growth of human colon organoid formation, while deletion of LYAR or NOTCH2 lost this function (Fig. 6F). In addition, deletion of *SNORA54* did not affect expression of LYAR but promoted expression of NOTCH2, whereas knockout of LYAR suppressed expression of NOTCH2 in human colon organoids (fig. S7, D and E). These data suggest that *SNORA54* is highly conserved that may exert a similar mechanism to human ISCs. Together, these results indicate that *Snora54* negatively regulates the self-renewal of ISCs via suppression of Notch2 signaling pathway.

## DISCUSSION

Intestinal epithelial cells are among the fastest renewing cells in the body, with an average turnover rate of 3 to 5 days. ISCs can sense stemness signals and maintain the rapid turnover of intestinal epithelial cells through continuous self-renewal, thereby supporting epithelial regeneration and sustaining homeostasis in the intestinal system (*13*, *35*). Notably, epigenetic regulation plays a critical role in the self-renewal of ISCs (*36*). We previously defined several noncoding RNAs that can regulate the self-renewal of ISCs (*37*–*39*). In this study, we identified a stem cell–associated snoRNA *Snora54* that is highly expressed in the nucleolus of ISCs. *Snora54* deletion enhances the self-renewal capacity of ISCs and intestinal regeneration. Mechanistically, in a steady state, highly expressed *Snora54* anchors the nucleolar protein Lyar in the nucleolus of ISCs, preventing Lyar from translocation into the nucleoplasm. Thereby, Lyar fails to recruit on the Notch2 promoter region in the nucleoplasm to promote *Notch2* transcription, leading to suppression of ISC self-renewal. By contrast, with deletion of *Snora54*, Lyar translocates to the nucleoplasm of ISCs where it enriches on the Notch2 promoter region to initiate its transcription resulting in the activation of Notch2 signaling pathway for maintenance of ISC stemness.

snoRNAs are one of the most classical categories of noncoding RNAs. snoRNAs are classified into two types: CD box and H/ACA box. For their canonical functions, the C/D box snoRNAs mediate 2′-*O*-methylation of rRNAs (*40*, *41*), while the H/ACA box snoRNAs account for pseudouridylation of rRNAs (*42*, *43*). snoRNAs are primarily located in the nucleolus of the cell. Their nucleolar localization is directly related to their canonical functions: Most snoRNAs serve as guide RNAs that are involved in the posttranscriptional modification of rRNA and some spliceosome RNAs, while a few snoRNAs participated in the intranuclear processing of precursor rRNA transcripts (*44*, *45*). However, recent studies reported that some snoRNAs have noncanonical functions that are not associated with rRNA modifications. For example, *SNORA71A* promotes the development of breast cancer by up-regulating mRNA and protein levels of ROCK2 through its interaction with G3BP1 (*46*). *SNORA72* elevates mRNA and protein expression levels of Notch1 and c-Myc in parental cells, thereby activating the stemness transformation of ovarian cancer cells (*47*). In this study, we showed that *Snora54*-mediaed pseudouridylation of 28*S* rRNA does not affect organoid formation, indicating that *Snora54* regulates the ISC stemness with a noncanonical manner. We identified that highly expressed *Snora54* in ISCs anchors the nucleolar protein Lyar in the nucleolus to impede its translocation to the nucleoplasm in a steady state, leading to inhibition of enrichment of Lyar on the Notch2 promoter region for its transcription.

Lyar is a zinc-finger protein localized in both the nucleoplasm and the nucleolus, capable of binding to a specific DNA motif and exerting transcriptional regulatory functions (*23*). For instance, in erythroid progenitor cells, Lyar binds to Prmt5 at the promoter regions of Hbg1 and Hbg2 to inhibit their expression (*23*). Lyar directly interacts with the promoter region of Fscn1 to up-regulate its expression, thereby affecting the downstream fatty acid metabolism in colorectal cancer cells (*24*). In addition, Lyar can recruit Brd2 protein to chromatin, regulating the down-regulation of Nanog to ensure the proper establishment of differentiation programs (*25*). However, up to date, there have been no studies about the roles of Lyar in ISC biology. We found that Lyar is primarily localized in the nucleolus of ISCs that binds to *Snora54*, thereby keeping its nucleolar residency. With deletion of *Snora54*, Lyar translocates into the nucleoplasm to enrich on the Notch2 promoter region for initiation of its transcription. However, how *Snora54* mediates translocation still needs further investigation.

The self-renewal of ISCs is finely modulated by various signaling pathways. Within ISCs, Notch signaling works in conjunction with Wnt signaling to sustain the stem cell properties and guide the differentiation of ISCs (*26*, *48*, *49*). High levels of Notch signaling can promote the differentiation of ISCs into TA cells and absorptive cells, while inhibition of Notch signaling causes a sustained decrease in both ISCs and absorptive lineage cells, accompanied by an increase in secretory lineage cells (*27*–*29*). Therefore, a stable and appropriate level of Notch signaling is essential for maintaining intestinal homeostasis (*50*). Notch1 and Notch2 are the primary Notch receptors in ISCs, engaging Notch ligands secreted by Paneth cells to activate downstream signaling pathways (*51*). Here, we demonstrated that translocated Lyar binds to a specific DNA motif in the Notch2 promoter in *Snora54*-deficient ISCs, thereby activating transcription of *Notch2* leading to activation of Notch2 signaling pathway.

In summary, we identified an snoRNA *Snora54* that is highly expressed in the nucleolus of ISCs. In a steady state, *Snora54* binds Lyar protein to anchor it in the nucleolus, preventing its enrichment on the Notch2 promoter region from its transcription. Therefore, *Snora54* negatively regulates self-renewal of ISCs and gut regeneration via suppression of Notch2 signaling pathway. We might deplete *Snora54* expression via antisense oligos with a targeting delivery system by lipid nanoparticles to develop more effective therapeutic strategies for gut inflammation and regeneration.

## MATERIALS AND METHODS

### Antibodies and reagents

Anti-Notch2 (catalog no. 5732), anti–H3K27 acetylation (catalog no. 8173), and anti-H3K27me3 (catalog no. 9733) were purchased from Cell Signaling Technology. Anti-Ki67 (catalog no. AB15580), anti-Npm1 (catalog no. AB52644), and F-actin staining kit (catalog no. AB1112127) were all obtained from Abcam. Anti–β-actin (catalog no. A1978) and anti-Flag (catalog no. F1804) antibodies were from Sigma-Aldrich. Alexa Fluor 594–, Alexa Fluor 488–, and Alexa Fluor 647–conjugated anti-rabbit and anti-mouse secondary antibodies were purchased from Invitrogen. Anti-Nap1l4 (catalog no. 16018-1-AP) was purchased from Proteintech. Anti-Ncl (catalog no. bs-8536R) and anti-Lyar (catalog no. bs-18456R) were purchased from Bioss. The dual luciferase reporter gene assay kit (catalog no. RG027) was purchased from Beyotime. Biotin RNA labeling mix (catalog no. 11685597910) and T7 RNA polymerase (catalog no. 10881767001) were from Roche. Paraformaldehyde (PFA) and 4′,6-diamidino-2-phenylindole (DAPI) were from Sigma-Aldrich. DNase I was purchased from Roche Molecular Biochemicals (Basel, Switzerland).

### Cell lines and human samples

CT26 cell lines were provided by American Type Culture Collection. All the cell lines were maintained in Dulbecco’s modified Eagle’s medium (DMEM) supplemented with 10% fetal bovine serum, penicillin G (100 μg/ml), and streptomycin (100 U/ml). Human colon organoids are cultured from the peritumoral tissues of consenting colorectal cancer patients at the Peking University Third Hospital. Human colon samples were treated within 2 hours after resection. All studies using human samples were conducted in accordance with relevant guidelines and received approval from the Institutional Review Board of the Institute of Biophysics, Chinese Academy of Sciences (SYXK2021078).

### Generation of *Snora54* knockout mice

To generate *Snora54* knockout (*Snora54*^−/−^) mice, a CRISPR-mediated approach was used as previously described (*38*). A pair of single guide RNAs (sgRNAs) targeting the intron sequences flanking the *Snora54* locus was designed. The corresponding sgRNAs are detailed in table S1. Approximately 250 zygotes from C57BL/6 mice were injected with the sgRNAs and subsequently transferred to the uteri of pseudopregnant ICR females, resulting in the birth of viable founder mice. Genomic DNA mutation was identified through PCR screening and DNA sequencing, followed by Northern blot analysis. All mice were backcrossed into the C57BL/6 genetic background for a minimum of 10 generations. Both male and female mice aged between 2 and 4 months were used in the experiments. No animals were excluded from the study, and the subjects were not randomized. The investigators were not blinded to the allocation during the experiments and outcome assessments. For irradiation, the mice received a single dose of abdominal x-ray radiation (8 Gy) and were subsequently analyzed at various time points. All animal studies were conducted in accordance with relevant guidelines and received approval from the Institutional Animal Care and Use Committees at the Institute of Biophysics, Chinese Academy of Sciences (SYXK2021071).

### CRISPR-Cas9 knockout system

*Snora54*, Lyar, and Notch2 deletion organoids were generated using CRISPR-Cas9 technology provided by Zhang’s laboratory (*52*). All sgRNAs were designed by online CRISPR design tool (https://design.synthego.com), with the specific sgRNA sequences detailed in table S1. Briefly, the sgRNAs were cloned into LentiCRISPRv2 vector (catalog no. 52961, Addgene). LentiCRISPRv2, pVSVg (catalog no. 8454, Addgene), and psPAX2 (catalog no. 12260, Addgene) plasmids are used to produce CRISPR-Cas9 lentiviral particles. Lentiviral vectors and packaging plasmids were cotransfected into human embryonic kidney (HEK) 293T cells using Lipofectamine 3000 for a duration of 48 hours. Following transfection, the supernatant was filtered through 0.45-μm filters and concentrated using Lenti-Concentin Virus Precipitation Solution (Genstar). Organoids were infected at 37°C for 12 hours, followed by selection with puromycin. The organoid cells were expanded and validated through DNA electrophoresis and sequencing.

### shRNA knockdown system

Target genes were silenced using shRNA as previously described (*53*). All shRNAs were designed using the BLOCK-iT RNAi Designer (Thermo Fisher Scientific). Specific shRNAs for each indicated gene were selected and cloned into the pSicoR-Puro lentiviral vector (catalog no. 12084, Addgene), with corresponding primers listed in table S4. Recombinant pSicoR-Puro vectors were cotransfected with packaging plasmids pVSVg (catalog no. 8454, Addgene) and psPAX2 (catalog no. 12260, Addgene) into HEK293T cells using Lipofectamine 3000 for a duration of 48 hours. Following transfection, the supernatant was filtered through 0.45-μm filters and concentrated using Lenti-Concentin Virus Precipitation Solution (Genstar). The concentrated virus was then mixed with an equal volume of fresh DMEM, and ISCs were infected at 37°C for 12 hours, followed by selection with puromycin. ISCs were passaged, and the efficiency of gene silencing was assessed using qRT-PCR.

### Isolation of intestinal crypts and organoid formation assay

For crypts isolation, mouse intestine was scraped with slides, cut into pieces, and washed six times with phosphate-buffered saline (PBS). These tissues were digested by 0.1% type I collagenase (Invitrogen) for about 20 min. The mixture obtained after washing and repeatedly blowing was then passed through 70-mm cell strainer (BD Biosciences) and centrifuged at 70*g* for 5 min to collect intestine crypts. For organoid culture, crypts were embedded in Matrigel (BD Biosciences) and seeded on six-well plate. After polymerization, IntestiCult Organoid Growth Medium (STEMCELL Technologies) was added and refreshed every 3 days. For passaging, the medium was removed and ice-cold PBS was added to melt the gel. Then, organoids were pelleted by centrifugation at 70*g* for 5 min, embedded in fresh Matrigel, and seeded on new plate with fresh medium added.

### Isolation of nucleolus, nucleoplasm, and cytoplasm

The isolation of nucleolus was performed according to the protocol (*54*). Briefly, the collected cells were lysed in Nuclear Separation Buffer and 0.3% NP-40, and the supernatant obtained after centrifugation (4°C for 5 min at 1200*g*) represented the cytoplasmic fraction. The purified nuclei were disrupted under sonication, and the nucleoplasm and nucleolar components were separated by sucrose gradient centrifugation.

### EdU incorporation assay

EdU solution was added to cultured organoids to a final concentration of 10 μM and incubated 2 hours, and then EdU was detected by the BeyoClick EdU Cell Proliferation Kit according to the manufacturer’s protocol (catalog no. C0078S, Beyotime).

### Real-time quantitative PCR

Total RNA was extracted from samples and purified by TRIzol method. Reverse transcription was performed using 5× All-In-One RT Master Mix (Applied Biological Materials Inc.). Then, real-time PCR was performed using cDNA obtained above, SuperReal Premix Plus (SYBR Green) (TIANGEN), and corresponding primer (table S2).

### Northern blotting assay

Total RNA was extracted with TRIzol method and then subjected to electrophoresis on urea-polyacrylamide gel for 1 hour. Samples were transferred to positively charged nylon membranes with 0.5× tris-borate EDTA buffer. Membranes were cross-linked under 265-nm ultraviolet with an energy of 240,000 μJ/cm^2^. After prehybridization, membranes were incubated with biotin-labeled probes at 65°C overnight. After blocking and washing, biotin signals were detected with Chemiluminescent Nucleic Acid Detection Module (catalog no. 89880, Thermo Fisher Scientific) according to the manufacturer’s instructions.

### CMC treatment

Twenty micrograms of purified RNA was treated with 20 μl of 5 mM EDTA at 80°C for 3 min, then added 100 μl of BEU buffer (50 mM bicine, 4 mM EDTA, and 7 M urea), and incubated at 37°C for 30 min. Then, 500 μl of isopropanol was added, and the mixture was stored overnight at −20°C to precipitate the RNA. The extracted RNA was then resuspended in 30 μl of alkaline treatment solution (50 mM Na_2_CO_3_ and 2 mM EDTA) and incubated at 50°C for 2 hours. The extracted RNA was subjected to qRT-PCR analysis.

### Biotin-labeled RNA pull-down and mass spectrometry

The 5′-monophosphorylated linear probe of *Snora54* was in vitro transcribed using biotin RNA labeling mix (catalog no. 11685597910, Roche) and T7 RNA polymerase (catalog no. 108817670010, Roche). The lysates of crypt cells were incubated with biotin-labeled probes overnight and treated with 100 μl of streptavidin magnetic beads (BioLabs) for 4 hours. After washing with radioimmunoprecipitation assay (RIPA) buffer, enriched proteins were separated by SDS–polyacrylamide gel electrophoresis (PAGE) and visualized by silver staining. Different bands were cut and collected for mass spectrometry analysis (Q-Exactive, Thermo Fisher Scientific).

### Fluorescence in situ hybridization

Organoids or tissue slices were fixed with 4% PFA and incubated with FISH hybridization buffer [50% formamide, 2× SSC, yeast transfer RNA (0.5 mg/ml), salmon sperm DNA (0.5 mg/ml), and bovine serum albumin (2.5 mg/ml)] and probe overnight. Fluorescent in situ hybridization kit (RiboBio) was used for subsequent processing. Samples were observed by confocal microscopy (Nikon A1R+).

### Immunofluorescence staining

Samples were fixed by 4% PFA for 20 min and permeated by 1% Triton X-100 for 15 min. After blocking with 10% donkey serum for 20 min, primary antibodies were added and incubated overnight at 4°C. Fluorescence-conjugated secondary antibodies were added after washing three times with PBS and incubated at room temperature for 1 hour. After sealing, confocal microscopy (Nikon A1R+) was performed for observation.

### Immunoblotting

Cells, crypts, or organoids were harvested, washed twice with cold PBS, and lysed with RIPA lysis buffer (strong) (GenStar). Lysates were centrifuged at 14,000*g* for 20 min at 4°C. The protein was separated with SDS-PAGE, transferred to nitrocellulose membrane (Bio-Rad), and incubated with primary antibodies overnight. After washing three times with tris-buffered saline with Tween 20, membranes were incubated with horseradish peroxidase–conjugated secondary antibodies for 1 hour at room temperature for visualization.

### ChIP assay

ChIP assay was performed using the BeyoChIP Enzymatic ChIP Assay Kit (catalog no. P2083S, Beyotime). Briefly, ISCs were fixed in 1% formaldehyde at 37°C for 10 min. The collected cells were lysed and treated with micrococcal nuclease to shear DNA into fragments between 150 and 1000 bp. The samples were precleared using Protein A/G Magnetic Beads for 30 min in rotor, followed by incubation with anti-Lyar (catalog no. bs-18456R) at a dilution of 1:100. The eluted fractions were subsequently subjected to DNA isolation and analyzed via qRT-PCR. The primer sequences are listed in table S3.

### DNase I accessibility assay

Cell nuclei were isolated from organoids according to the protocol from Nuclei Isolation Kit (Sigma-Aldrich). Then, cell nuclei were digested with 200 μl of DNase I digestion buffer (1 mM EDTA, 0.1 mM EGTA, 5% sucrose, 1 mM MgCl_2_, and 0.5 mM CaCl_2_) for 5 min at 37°C. After stopping by 2× DNase I stop buffer [20 mM tris (pH 8.0), 4 mM EDTA, and 2 mM EGTA], total DNA was extracted and followed by qRT-PCR.

### Statistics

For statistical analysis, data were analyzed with unpaired Student’s *t* test using GraphPad Prism 8.0.2. *P* ≤ 0.05 was considered significant (*P ≤ 0.05, **P < 0.01, and ***P < 0.001), and *P* > 0.05 was considered nonsignificant (ns).
