# Supplementary material for: Snora54 negatively regulates self-renewal of intestinal stem cells and gut regeneration via suppression of Notch2 signaling
Source: Sci Adv. 2025 May 23;11(21):eadv4725. doi: 10.1126/sciadv.adv4725 (PMC12101510; doi:10.1126/sciadv.adv4725)
Supplement: Supplementary file 1 — Figs. S1 to S7 Tables S1 to S4 [file sciadv.adv4725_sm.pdf]

Science Advances
AAAS

# Supplementary Materials for

## *Snora54* negatively regulates self-renewal of intestinal stem cells and gut regeneration via suppression of Notch2 signaling

Jiahang Zhang *et al.*

Corresponding author: Pingping Zhu, ppzhustc@126.com; Zusen Fan, fanz@moon.ibp.ac.cn

*Sci. Adv.* **11**, eadv4725 (2025)
DOI: 10.1126/sciadv.adv4725

**This PDF file includes:**

Figs. S1 to S7
Tables S1 to S4

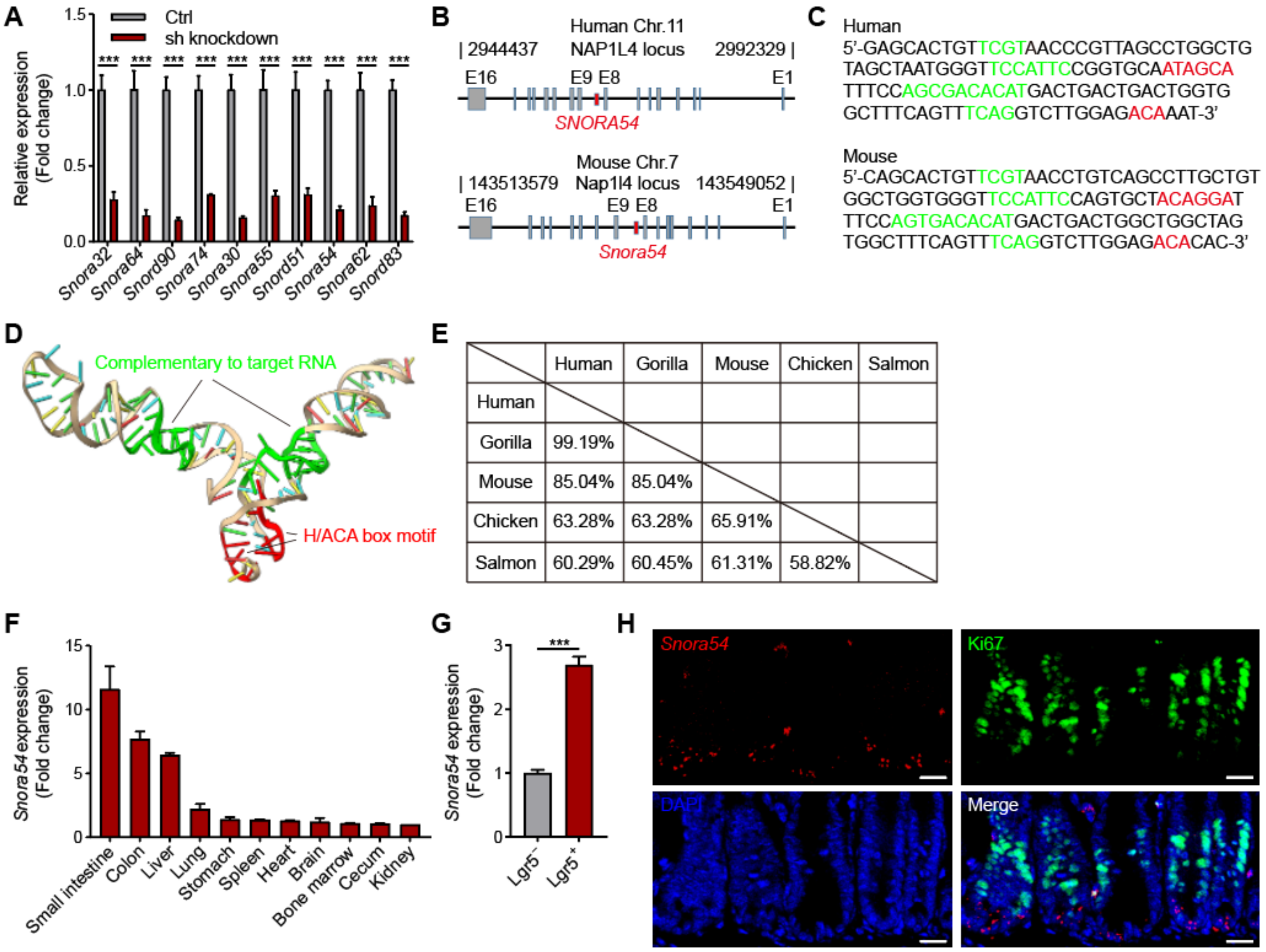


**Figure S1. Characterization and localization of *Snora54*.** (A) Knockdown efficiencies were detected by qRT-PCR. n = 3. Results are shown as means ± SD. ***$P$ < 0.001 by two-tailed Student's t-test. (B) Illustration of genomic region of *Snora54* and host gene *Nap1l4* in human and mice. (C) Sequences of *Snora54* in human and mice. Green region shows motifs complementary to target RNA, red region shows H/ACA box motifs. (D) Predicted tertiary structure of *Snora54*, visualized by ChimeraX 1.8. Green region shows motifs complementary to target RNA, red region shows H/ACA box motifs. (E) Sequence conservation analysis of *Snora54* in vertebrates from salmon to human. (F) Expression levels of *Snora54* in different tissues were detected by qRT-PCR. n = 3 independent experiments. Results are shown as means ± SD. (G) Expression levels of *Snora54* in Lgr5$^-$ and Lgr5$^+$ cells were detected by qRT-PCR. n = 3 independent experiments. Results are shown as means ± SD. ***$P$ < 0.001 by two-tailed Student's t-test. (H) RNA fluorescence in situ hybridization of *Snora54* in small intestine tissues. Scale bars, 20 μm.

Zhang J, et al. Figure S2

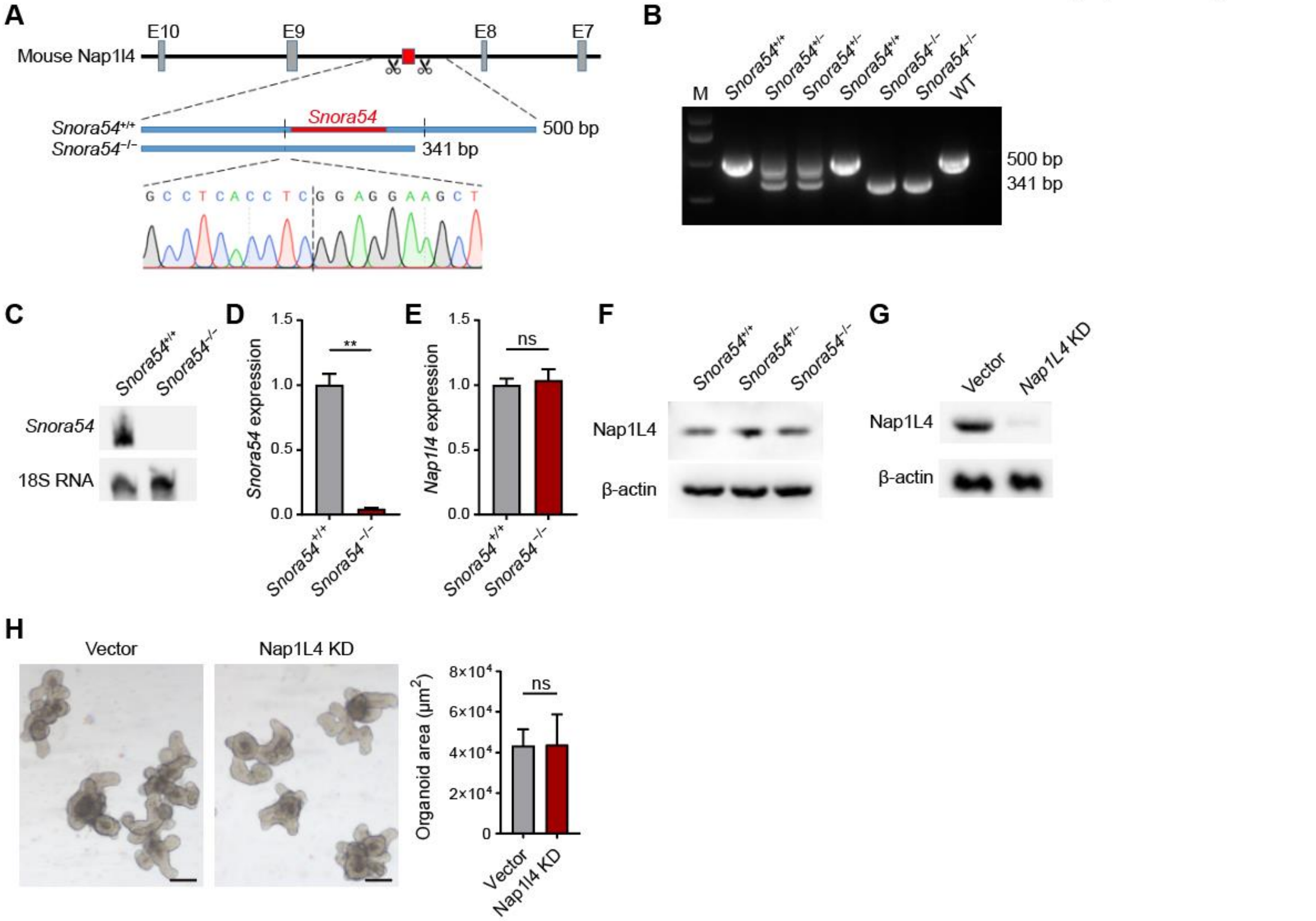


**Figure S2. Validation of *Snora54*$^{-/-}$ mice and detection of self-renewal maintenance of Nap1l4 depletion.** (A) Scheme for *Snora54* knockout mice. Intronic Sequences were deleted through CRISPR/Cas9 approach. (B) *Snora54*$^{-/-}$ mice were identified by agarose gel electrophoresis. (C) Northern blot analysis for *Snora54* expression in mouse ISCs. 18S RNA served as a loading control. (D) Expression levels of *Snora54* in *Snora54*$^{+/+}$ and *Snora54*$^{-/-}$ mice ISCs were detected by qRT-PCR. n = 3 independent experiments. Results are shown as means ± SD. ***P* < 0.01 by two-tailed Student's t-test. (E) Expression levels of *Nap1l4* in *Snora54*$^{+/+}$ and *Snora54*$^{-/-}$ mice ISCs were detected by qRT-PCR. n = 3 independent experiments. Results are shown as means ± SD. NS, not significant by two-tailed Student's t-test. (F) Immunoblotting analysis of Nap1l4 expression in *Snora54*$^{+/+}$, *Snora54*$^{+/-}$ and *Snora54*$^{-/-}$ mice ISCs. (G) Immunoblotting analysis of Nap1l4 expression in *Nap1l4* knockdown (*Nap1l4* KD) from WT mice organoid. (H) Organoid formation was conducted with *Nap1l4* knockdown (*Nap1l4* KD) from WT mice organoid, vector served as control. Scale bars, 100 μm. Organoid areas per field (n = 3) are shown as means ± SD. NS, not significant by two-tailed Student's t-test.

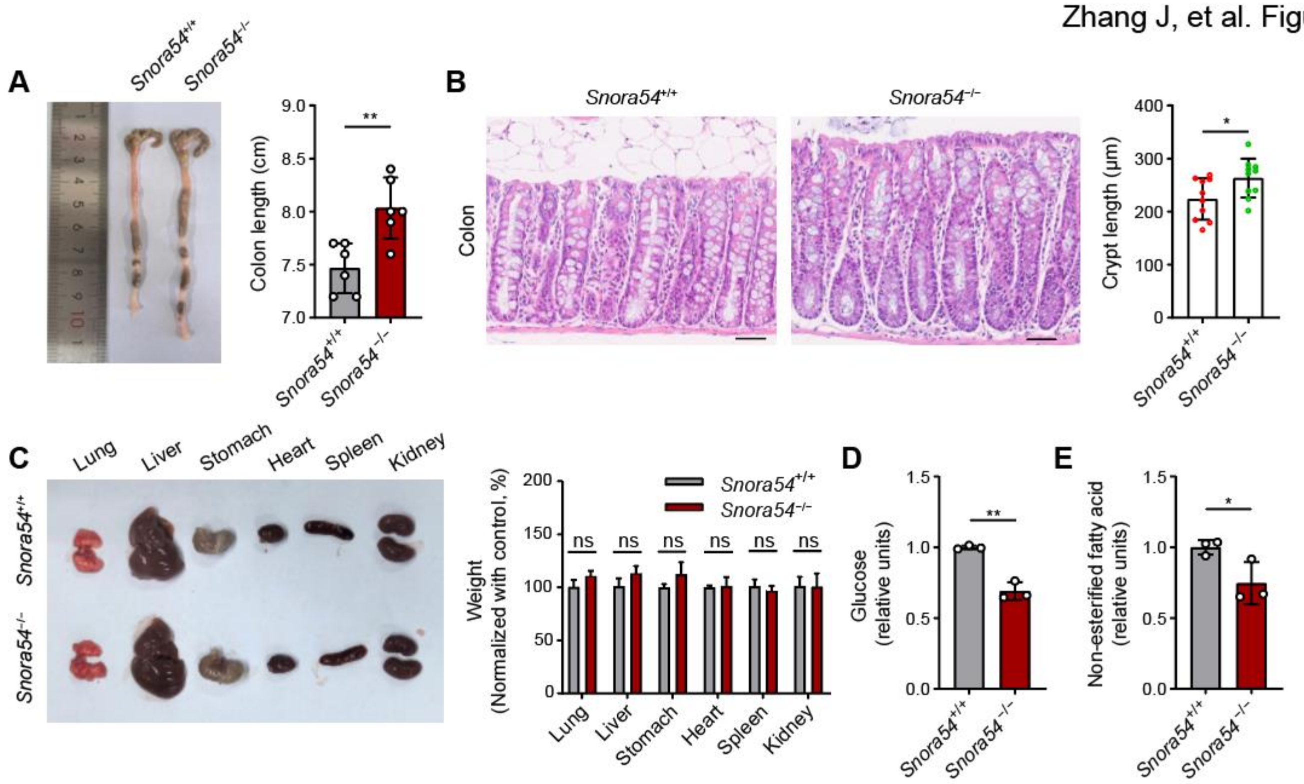


**Figure S3. Deletion of *Snora54* promotes self-renewal of ISCs and intestinal absorption.** (A) Colon image of *Snora54*$^{+/+}$ and *Snora54*$^{-/-}$ mice. Colon lengths (n = 6) are shown as means ± SD. ***P* < 0.01 by two-tailed Student's t-test in right panel. (B) H&E staining of colon from *Snora54*$^{+/+}$ and *Snora54*$^{-/-}$ mice. Average length of crypts in a visual field (n = 10 fields) from *Snora54*$^{+/+}$ and *Snora54*$^{-/-}$ mice are shown. Length of crypts were calculated as means ± SD. **P* < 0.05 by two-tailed Student's t-test in right panel. Scale bars, 50 μm. (C) Different tissue images of *Snora54*$^{+/+}$ and *Snora54*$^{-/-}$ mice. Weight measurements of stomachs removed its contents. Results are shown as means ± SD. NS, not significant by two-tailed Student's t-test. (D, E) Faeces from *Snora54*$^{+/+}$ and *Snora54*$^{-/-}$ mice raised under similar conditions were collected and dried. Levels of glucose (D) and non-esterified fatty acid (E) in extracts were measured. n = 3 independent experiments. Results are shown as means ± SD. **P* < 0.05, ***P* < 0.01 by two-tailed Student's t-test.

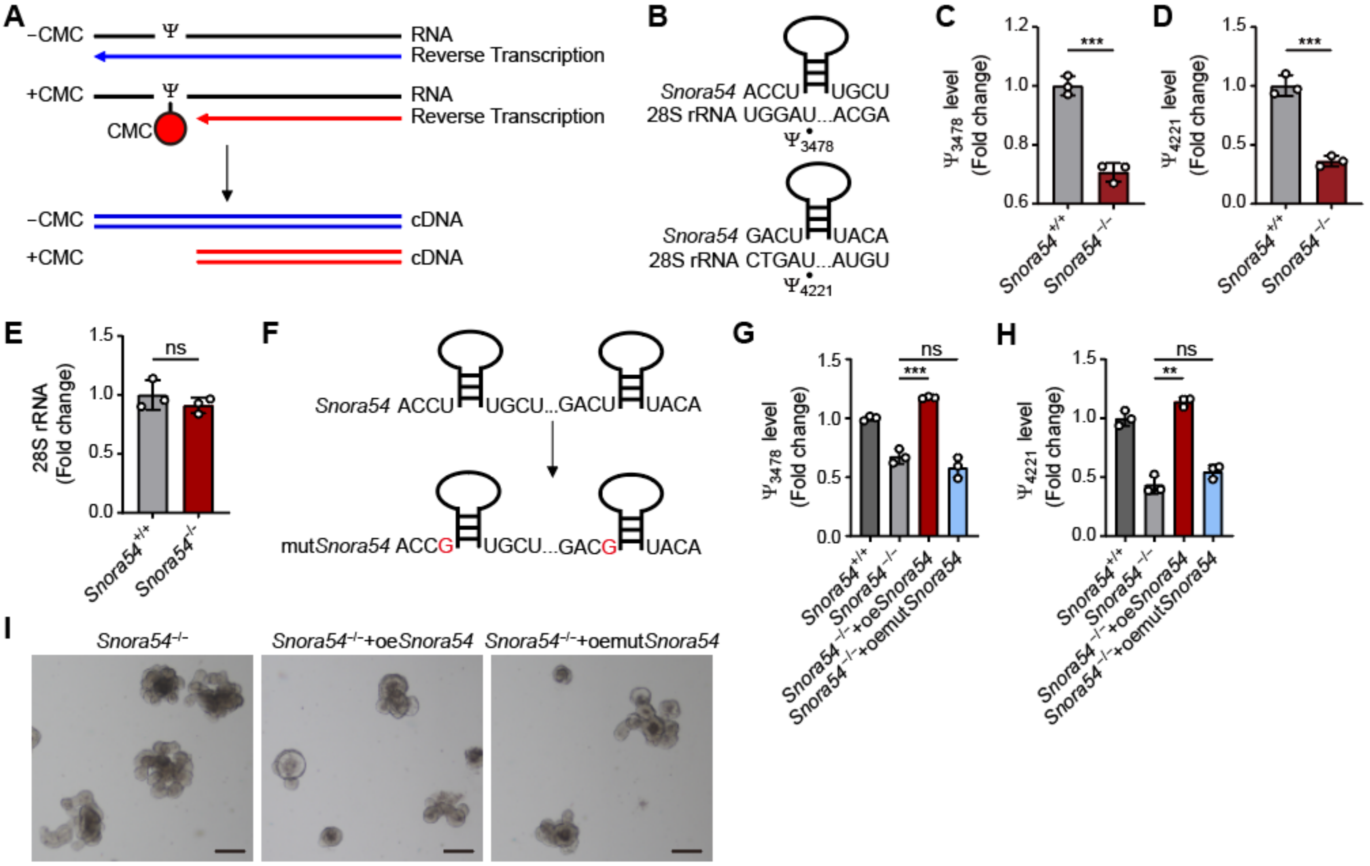


**Figure S4. Disruption of classic function of *Snora54* does not affect ISC stemness.** (A) Diagram for determining pseudouridylation levels at specific sites using CMC method. (B) Binding of *Snora54* with 28S rRNA and pseudouridine modification sites. (C, D) Levels of pseudouridylation at site 3478 (C) and site 4221 (D) in *Snora54*$^{+/+}$ and *Snora54*$^{-/-}$ mice ISCs were detected by qRT-PCR. n = 3 independent experiments. Results are shown as means ± SD. ****P* < 0.001 by two-tailed Student's t-test. (E) Levels of 28S rRNA in *Snora54*$^{+/+}$ and *Snora54*$^{-/-}$ mice ISCs were detected by qRT-PCR. n = 3 independent experiments. Results are shown as means ± SD. NS, not significant by two-tailed Student's t-test. (F) Sequences of *Snora54* mutant. (G, H) Levels of pseudouridylation at site 3478 (G) and site 4221 (H) in *Snora54*$^{+/+}$ and *Snora54*$^{-/-}$ organoids with *Snora54* overexpression and *Snora54* mutant overexpression were detected by qRT-PCR. n = 3 independent experiments. Results are shown as means ± SD. NS, not significant, ***P* < 0.01 by two-tailed Student's t-test. (I) Organoid formation was conducted from *Snora54*$^{-/-}$ mice ISCs with *Snora54* overexpression or *Snora54* mutant overexpression. Scale bars, 100 μm.

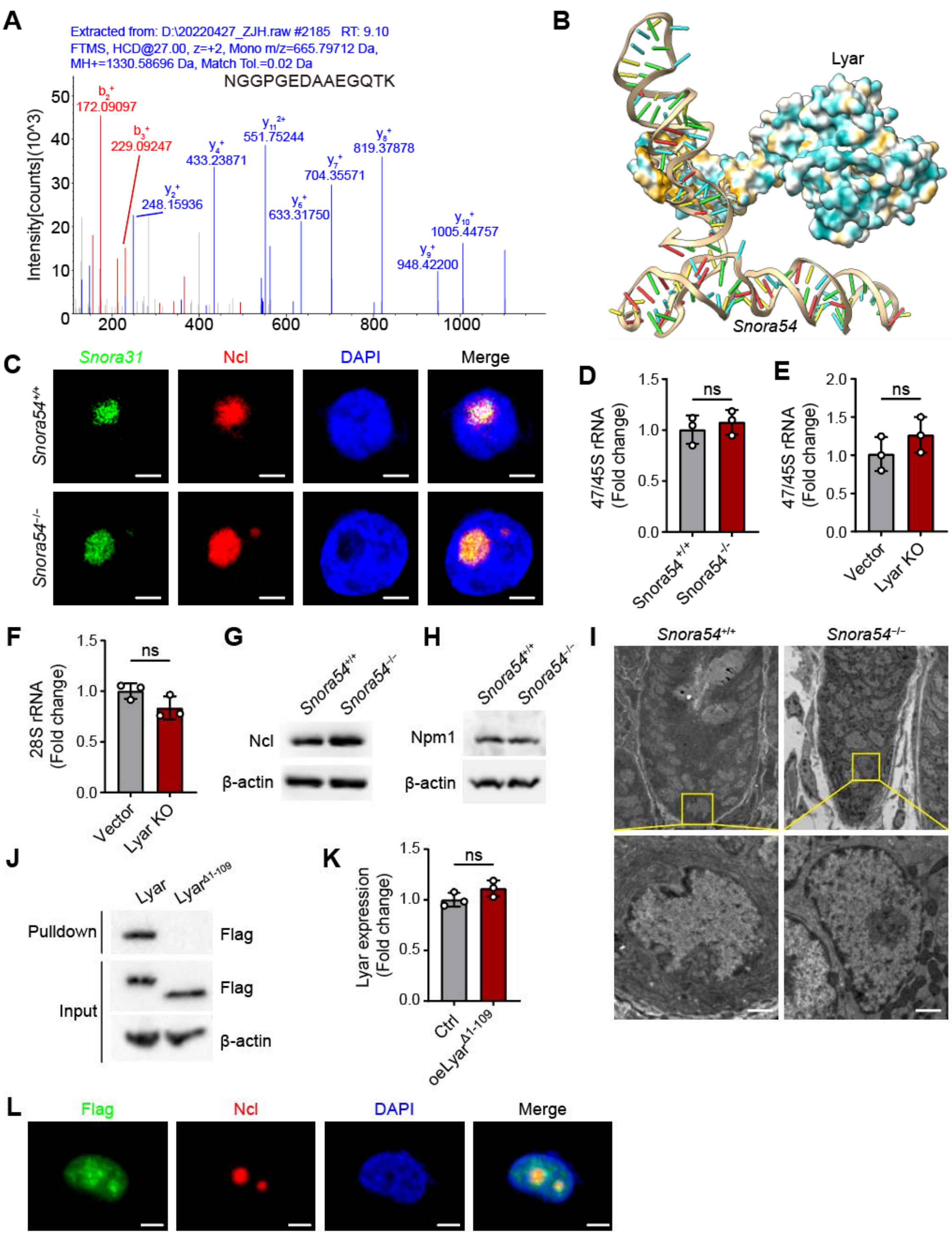


**Figure S5. *Snora54* interacts with Lyar in ISCs.** (A) MS profiles of Lyar, corresponding peptide sequences were listed on top of diagram. (B) Three-dimensional docking prediction diagram of *Snora54* and Lyar was analyzed by HDOCK server (http://hdock.phys.hust.edu.cn/), and then visualized by ChimeraX 1.8. (C) RNA fluorescence in situ hybridization of *Snora31* in *Snora54*$^{+/+}$ and *Snora54*$^{-/-}$ ISCs. Scale bar, 5 μm. (D, E) Levels of 47S/45S rRNA in *Snora54*$^{-/-}$ mice ISCs (D) and Lyar KO organoids (E) were detected by qRT-PCR. n = 3. Results are shown as means ± SD. NS, not significant by two-tailed Student's t-test. (F) Levels of 28S rRNA in Lyar KO organoids were detected by qRT-PCR. n = 3. Results are shown as means ± SD. NS, not significant by two-tailed Student's t-test. (G, H) Immunoblotting analysis of Ncl (G) and Npm1 (H) in *Snora54*$^{+/+}$ and *Snora54*$^{-/-}$ ISCs. (I)

Electron microscope images of *Snora54*$^{+/+}$ and *Snora54*$^{-/-}$ mouse crypts. Scale bars, 2 μm. (J) Full length Lyar (Lyar$^{FL}$) and truncated Lyar missing predicted binding domain (Lyar$^{\Delta 1\text{-}109}$) were incubated with *Snora54*, followed by RNA pulldown assay and Western blotting. (K) Expression levels of Lyar under truncated Lyar overexpression in mouse ISCs were detected by qRT-PCR. n = 3. Results are shown as means ± SD. NS, not significant by two-tailed Student's t-test. (L) Immunobloting of Flag-tagged truncated Lyar in mouse ISCs. Scale bar, 5 μm.

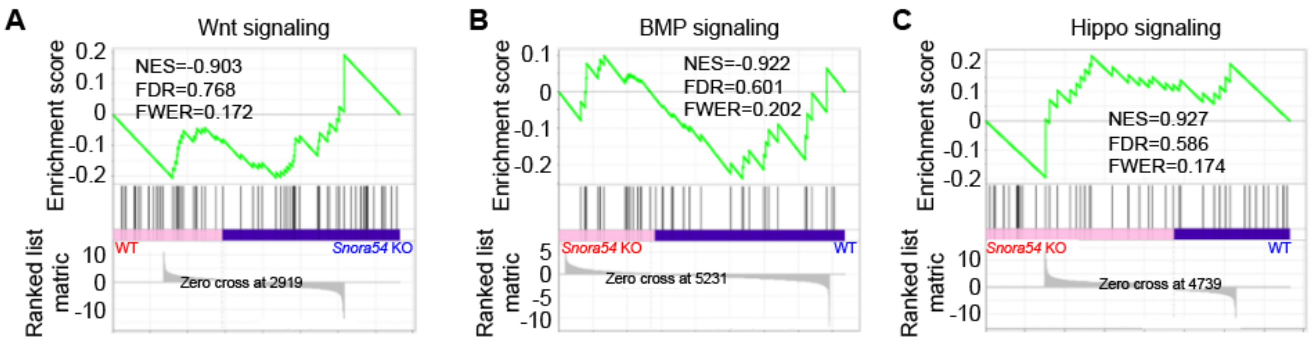


**Figure S6. GSEA analysis.** (A-C) GSEA analysis of Wnt (A), BMP (B) and Hippo (C) signaling pathways in *Snora54* KO and WT ISCs.

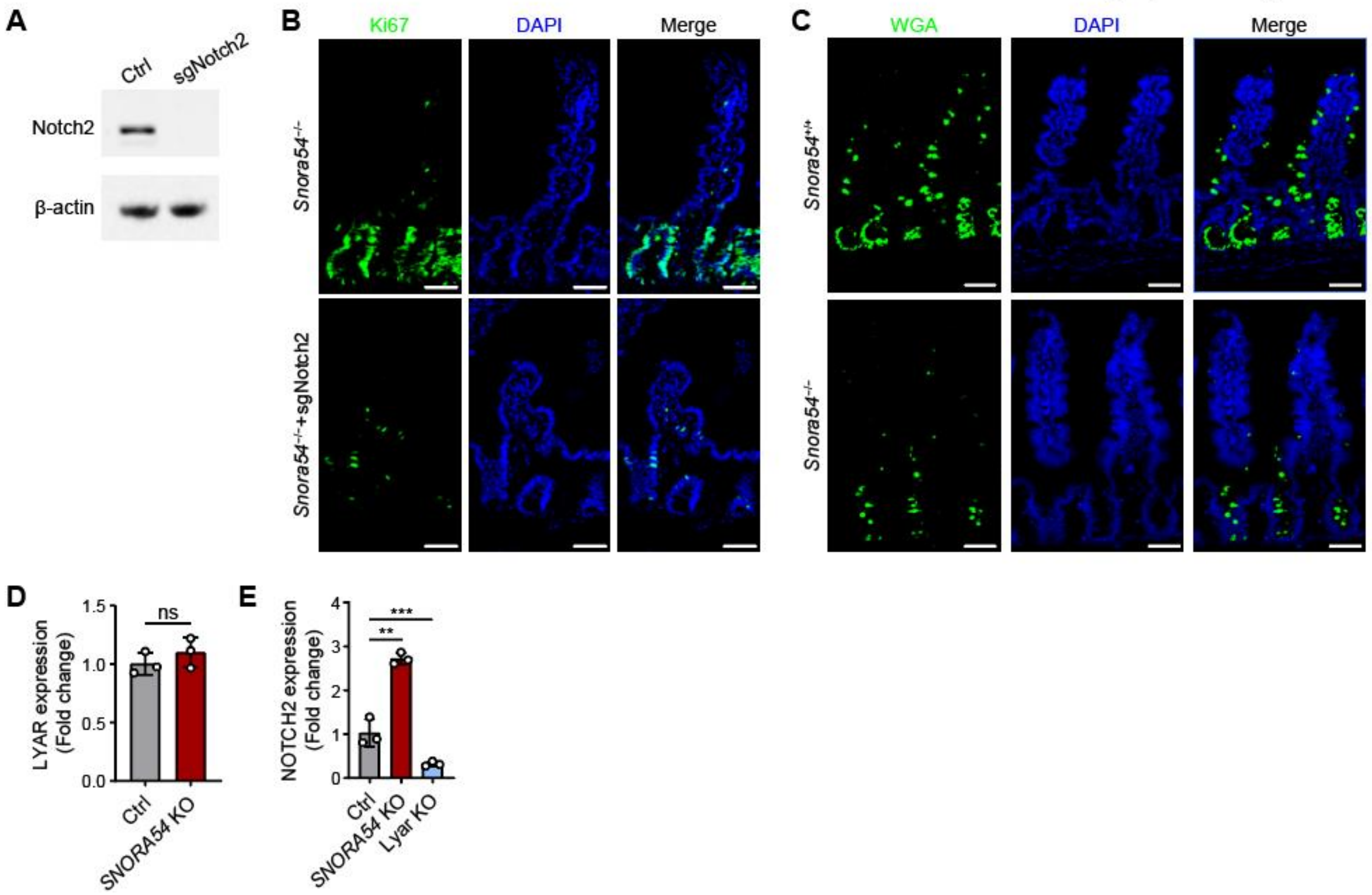


**Figure S7. Identification of Notch2 deletion and proliferation effect of Notch2.** (A) Immunoblotting analysis of Notch2 expression in sgScramble control (Ctrl) and sgNotch2 mouse ISCs. (B) Representative images of Ki67 staining of small intestine from *Snora54*$^{-/-}$ and *Snora54*$^{-/-}$;sgNotch2 mice. Scale bars, 50 μm. (C) Representative images of WGA staining of small intestines from *Snora54*$^{+/+}$ and *Snora54*$^{-/-}$ mice. Scale bars, 50 μm. (D) Expression levels of LYAR in *SNORA54* KO human colon organoids were detected by qRT-PCR. n = 3. Results are shown as means ± SD. NS, not significant by two-tailed Student's t-test. (E) Expression levels of NOTCH2 in *SNORA54* KO and LYAR KO human colon organoids were detected by qRT-PCR. n = 3. **$P$ < 0.01, ***$P$ < 0.001 by two-tailed Student's t-test.

**Table S1. sgRNAs used for CRISPR/Cas9-mediated mouse construction and *in vitro* deletion in this study**

| Genes | sgRNA sequences |
|---|---|
| *Snora54* mice (up) | 5'-CACCTTCTACTCAGCACTGT-3' |
| *Snora54* mice (down) | 5'-TAGGCAAGGGATCTTGACGG-3' |
| *Snora54* #1 (up) | 5'-CTTCACTGATGCACTTCACA-3' |
| *Snora54* #1 (down) | 5'-TGTGAAGTGCATCAGTGAAG-3' |
| *Snora54* #2 (up) | 5'-TACTTCTGACCTTCACTGAT-3' |
| *Snora54* #2 (down) | 5'-TCCCAGCCCTTGGGAGGCAG-3' |
| *Lyar* (up) | 5'-AGCCTCAAAGAGAAAAGGGC-3' |
| *Lyar* (down) | 5'-ACCTTCTTAATACAGATGTCCAG-3' |
| *Notch2* (up) | 5'-AACCTGGACATCTGTATTAAGAA-3' |
| *Notch2* (down) | 5'-ATCAGTGAAGGTCAGAAGTA-3' |

**Table S2. Primer oligonucleotides and probes used in this study**

| Genes | Forward | Reverse |
|---|---|---|
| *Snora54* #1 | 5'-AGCACTGTTCGTAACCTGTC-3' | 5'-AAGACCTGAAACTGAAAGCCAC-3' |
| *Snora54* #2 | 5'-CGTAACCTGTCAGCCTTGCT-3' | 5'-AGACCTGAAACTGAAAGCCACT-3' |
| *Snora54* #3 | 5'-TAACCTGTCAGCCTTGCTGTG-3' | 5'-AGACCTGAAACTGAAAGCCAC-3' |
| 18S | 5'-TAACGAACGAGACTCTGGCAT-3' | 5'-CGGACATCTAAGGGCATCACAG-3' |
| *Actb* | 5'-GTCACCAACTGGGACGACAT-3' | 5'-AGGGATAGCACAGCCTGGAT-3' |
| *Gapdh* | 5'-AGGTCGGTGTGAACGGATTTG-3' | 5'-TGTAGACCATGTAGTTGAGGTCA-3' |
| *U6* | 5'-GCTTCGGCAGCACATATACTAAAAT-3' | 5'-CGCTTCACGAATTTGCGTGTCAT-3' |
| *U8* | 5'-ACCTGTTCCTCGTTTTGGAG-3' | 5'-GGGTGTTGCAAGTCCTGATT-3' |
| *Nap1l4* | 5'-GGCCGACCCGTTTTCTTTTG-3' | 5'-TGGTGATGGTTCGTACGGTG-3' |
| *Nap1l4* intron8 | 5'-GGACAACACTGGTTCAGAAATCCA-3' | 5'-CGTGAACAAGAGGCAATCTTCA-3' |
| *Lyar* #1 | 5'-GAAGTACGGAGGCAAAGGCT-3' | 5'-TGCTGCAAAAGTTCTCGCAC-3' |
| *Lyar* #2 | 5'-ATGCAAAACAGCAGGCATGG-3' | 5'-CTGCTCTAGAACGGAGTCGC-3' |
| *Lyar* #3 | 5'-TCCTGCATTGACTGTGGGAAA-3' | 5'-ATCCATGCCTGCTGTTTTGC-3' |
| *Egfl7* | 5'-CTCGGTCCACAGAGCATGG-3' | 5'-CAGTGAATCAATTCGGTCCAGC-3' |
| *Postn* | 5'-TCCTAAATACCCTCCAGTGCT-3' | 5'-CCCTTCGCACCCTATCTCAAT-3' |
| *Enho* | 5'-ATCTCCCAAGGGGCTCTCAT-3' | 5'-CGACGTCAGCAGATCGAGAA-3' |
| *Bmp7* | 5'-CCATCTTAGGGTTGCCCCAT-3' | 5'-AGACGGCCTTGTAGGGGTAG-3' |
| *Hes1* | 5'-TGCCTTTCTCATCCCCAACG-3' | 5'-ACATGGAGTCCGAAGTGAGC-3' |
| *Hey2* | 5'-GGCTACTTTGATGCCCATGC-3' | 5'-GAGATGAGAGACAAGGCGCA-3' |
| *Sorbs2* | 5'-AATGACGACTGCGACAGCTT-3' | 5'-CCTAGATCTGCTGTTCCCGC-3' |
| *Niban2* | 5'-CCAACGTGGCAAGGAAAAGG-3' | 5'-TCACTCCCCTGTAAGACCCC-3' |
| *Notch1* | 5'-TGTGGCTTCCTTCTACTGCG-3' | 5'-CTTTGCCGTTGACAGGGTTG-3' |
| *Notch2* #1 | 5'-CAGCATGCGCTGTCTTTCTC-3' | 5'-GTAACCTGCCCAAGCTTCCT-3' |
| *Notch2* #2 | 5'-TGGGCAGCTGCTGTCAATAA-3' | 5'-TCTCGGGCAGCAAGAAACAA-3' |
| *Notch2* #3 | 5'-GTGTGGACAAAGTCAACCGC-3' | 5'-TTCAGGCAGGGAGTACTGGA-3' |
| *Notch3* | 5'-TGTGCAAATGGAGGTCGGT-3' | 5'-CTGAACTCTGGCAAACGCCT-3' |
| *Notch4* | 5'-CAGGAGTGTGAATCGGAGGTT-3' | 5'-CCAGATTTCCTAGCCCCAGTC-3' |
| *28S* | 5'-TGCCATGGTAATCCTGCTC-3' | 5'-GGGCGGGATTCTGACTTAG-3' |
| *28S*-Ψ3478 | 5'-GTGTTGACGCGATGTGATTT-3' | 5'-TAGGGACAGTGGGAATCTCG-3' |
| *28S*-Ψ4221 | 5'-CGATGTCGGCTCTTCCTATC-3' | 5'-AGCCAAGCACATACACCAAA-3' |

**Table S3. Primers for ChIP assay in this study**

| Genes | Forward | Reverse |
|---|---|---|
| *Notch2* pro-2000~-1800 | 5'-TTGTCAGTAGAGCACTGGAG-3' | 5'-TTAATGCCATCTCTTTGTTT-3' |
| *Notch2* pro-1800~-1600 | 5'-CAGTGGGGTAGGAGTGTTATTC-3' | 5'-CTGCTTACCATCCATACAACAC-3' |
| *Notch2* pro-1600~-1400 | 5'-AATGTGACCGTTTGCAGTAAG-3' | 5'-TCCTCTGACCTCCACACACA-3' |
| *Notch2* pro-1400~-1200 | 5'-TCTCTAACTTATTCCCTGGTTGG-3' | 5'-ACGGGGACCTGAGTTCAAAT-3' |
| *Notch2* pro-1200~-1000 | 5'-GCACAGCAAGCACTCTTTCC-3' | 5'-GTCTTTATGGCCCAGGATCA-3' |
| *Notch2* pro-1000~-800 | 5'-TGTGTTACTTATGCAATTCCTCGT-3' | 5'-TGTATGGACGTTTGTGTGCAT-3' |
| *Notch2* pro-800~-600 | 5'-TTTGGTTTTACGGCTGACG-3' | 5'-AGACAGCAGGAGCTTGAAAC-3' |
| *Notch2* pro-600~-400 | 5'-TTTCAAGCTCCTGCTGTCTCT-3' | 5'-GGCATTTCGTTCATTCATCC-3' |
| *Notch2* pro-400~-200 | 5'-CTCTGCCTTCCGTTGACAGT-3' | 5'-GAAGCGCCCAACATCAAAC-3' |
| *Notch2* pro-200~0 | 5'-GGTGCTGCTCACTCAGATCC-3' | 5'-CCCGAAGTTTGGCTGAAAG-3' |
| *Notch2* pro0~+200 | 5'-GGAGCTCCAGGACTCAGC-3' | 5'-ACAGCCAGAGCCACAGCA-3' |

**Table S4. shRNA sequences used in this study**

| Genes | shRNA sequences |
|---|---|
| *Snora32* | 5'-GAGTTAACCTGTTTGGAATATT-3' |
| *Snora64* | 5'-GTTTGTTGTTCCTAGGAGTTTC-3' |
| *Snord90* | 5'-GCATCATTGATTGTCCTATTAT-3' |
| *Snora74* | 5'-GTTACACTGTTGGAAGAGTAAA-3' |
| *Snora30* | 5'-GCTGAGATGAAACCCTTGATTA-3' |
| *Snora55* | 5'-GCTATACTAGAGCAGAGGAAAT-3' |
| *Snord51* | 5'-GGCTGAGCTCCTGATGGATTT-3' |
| *Snora54* | 5'-GCATTCCAGTGCTACAGGATTT-3' |
| *Snora62* | 5'-GAGTGTGCTACATTGAATTTAT-3' |
| *Snord83* | 5'-GCCACATGATGTTTGCATTTG-3' |
| *Nap1l4* | 5'-GCTGGAGATATGAAGAATAAA-3' |
